# Roles of Annexin A1 Expression in Small Cell Lung Cancer

**DOI:** 10.3390/cancers17091407

**Published:** 2025-04-23

**Authors:** Ágnes Paál, David Dora, Ákos Takács, Christopher Rivard, Shivaun Lueke Pickard, Fred R. Hirsch, Brigitta Roskó, Peter Kiraly, Péter Ferdinandy, Zoltán V. Varga, Zoltan Lohinai, Anikó Görbe

**Affiliations:** 1 Department of Pharmacology and Pharmacotherapy, Semmelweis University, 1085 Budapest, Hungary; agnesmpaal@gmail.com (Á.P.); takacs.akos@phd.semmelweis.hu (Á.T.); peter.ferdinandy@pharmahungary.com (P.F.); varga.zoltan@semmelweis.hu (Z.V.V.); 2 Center for Pharmacology and Drug Research & Development, Semmelweis University, 1085 Budapest, Hungary; 3 HCEMM-SU Cardiometabolic Immunology Research Group, Department of Pharmacology and Pharmacotherapy, 1085 Budapest, Hungary; 4 MTA-SE Momentum Cardio-Oncology and Cardioimmunology Research Group, 1089 Budapest, Hungary; 5 Department of Anatomy, Histology and Embryology, Semmelweis University, 1085 Budapest, Hungary; dora.david@semmelweis.hu (D.D.); brigitta.rosko@gmail.com (B.R.); 6 Division of Medical Oncology, University of Colorado Anschutz Medical Campus, Aurora, CO 80045, USA; chris.rivard@cuanschutz.edu (C.R.); shivaun.luekepickard@cuanschutz.edu (S.L.P.); fred.hirsch@mssm.edu (F.R.H.); 7 Tisch Cancer Institute, Center for Thoracic Oncology, Mount Sinai Health System, New York, NY 10029, USA; 8 Translational Medicine Institute, Semmelweis University, 1085 Budapest, Hungary; peter.kiraly@torokbalintkorhaz.hu; 9 Pharmahungary Group, 6722 Szeged, Hungary

**Keywords:** small cell lung cancer, annexin A1, short hairpin RNA, tumor microenvironment

## Abstract

Background/Objectives: Small cell lung cancer (SCLC) is one of the malignancies with the worst prognosis, and there have been no major breakthroughs in its treatment for a long time. The majority of patients are diagnosed at the extensive stage, where the only option is chemotherapy, and even the addition of immune checkpoint inhibitors results in only modest benefits. The characterization of the molecular mechanisms behind therapy resistance has relevance in finding novel therapeutic approaches. Previous studies showed the possibility of annexin A1’s (ANXA1) involvement in the immunosuppressive tumor microenvironment in SCLC, and there are studies showing the direct effects of ANXA1 modulation on cancer cell aggressiveness. Methods: We aimed to characterize the roles of ANXA1 expression using publicly available transcriptomic data, the RNA-seq-based predictive algorithms EPIC and ESTIMATE, and immunohistochemistry on patient samples. For the in vitro studies, we silenced ANXA1 expression with short hairpin RNA in three SCLC cell lines, measured the growth rate with the trypan blue exclusion assay, assessed the chemosensitivity to cisplatin and etoposide with the Presto Blue^TM^ viability assay, and performed Western blots to assess changes in the levels of metabolic and mesenchymal markers and transcriptional drivers. Results: ANXA1-high tumors are associated with significantly increased immune infiltrates, stromality, and tumor-associated macrophages (TAMs). The ANXA1 protein is expressed on tumor cells and TAMs at the tissue level. ANXA1 silencing in H841 cells did not affect the growth rate; in SW1271 cells, shANXA1 cells grew significantly slower than shCTRL cells. Meanwhile, in H1048 cells, proliferation was significantly faster. Despite the different growth rates of the tested cell lines, ANXA1 silencing decreased the chemosensitivity to both cisplatin and etoposide in all three cell lines. Gene expression changes in mesenchymal markers, metabolic markers, dominant transcriptional drivers, and immune-relevant molecules were also characterized. Conclusions: This is the first comprehensive characterization of ANXA1 in SCLC to reveal its role in the tumor’s cell biology and the TME, aiming to boost further research in the field.

## Simple Summary

Small cell lung cancer is one of the cancers with the worst outcomes, and, despite much research, its treatment remains unresolved. Possible links have been found between the gene expression patterns and immunogenicity, and thus the therapeutic sensitivity, of small cell lung cancer. Higher expression of the annexin A1 gene has been associated with worse outcomes. Our aim was to characterize the possible roles of annexin A1 in small cell lung cancer using bioinformatical and cell culture methods. Our results suggest that the role of annexin A1 is very complex and may contribute to our understanding of this aggressive disease.

## 1. Introduction

Small cell lung cancer (SCLC) is one of the malignancies with the worst prognosis, and there have been no major breakthroughs in its treatment for decades. Treatment guidelines approach the disease as a single entity, with grouping based on the physical extent of the cancer to the limited stage or extensive stage (ES) [1]. Unlike in non-small cell lung cancer (NSCLC), where well-known oncogenic drivers (e.g., VEGF, KRAS, EGFR, ALK, ROS1) can be effectively targeted with specific inhibitors [2], personalized medicine is not yet reliable in SCLC [3]. Genomic profiling identified an extremely high mutation rate in SCLC due to the obligatory inactivation of TP53 and/or RB1 tumor suppressor genes [1]. The basis of treatment is still platinum-based combination chemotherapy in both stages of SCLC. Clinical trials showed statistically significant improvements in overall survival [4,5] with the addition of the immune checkpoint inhibitors atezolizumab or durvalumab to combination chemotherapy in ES-SCLC patients. Therefore, this combination is approved as the first-line treatment for extensive-stage patients [6]. However, the benefits fall short of those in NSCLC, and biomarkers, such as PD-L1 expression and the tumor mutational burden, do not correlate with response rates [7]. Therefore, a paradigm shift in therapeutic approaches and basic research on SCLC’s molecular pathomechanisms and therapy resistance are warranted.

A promising approach to improving the SCLC therapy options, on which research has been focusing for the past decade, is its classification into molecular subtypes, instead of considering the disease as a single entity. Subclassifications have identified mutually broadly compatible characteristics, such as lower neuroendocrine (NE) marker expression (i.e., NE-low tumors) and stronger immune infiltration (i.e., immune-oasis tumors), with the dominant transcriptional drivers being POU domain class 2, transcription factor 3 (POU2F3) and yes-associated protein 1 (YAP1) [8,9]. Subtype heterogeneity and transition to the non-neuroendocrine state have also been proposed as markers of the progression of SCLC [8,10,11], but the underlying mechanisms are poorly understood. To find possible novel therapeutic approaches, research has shifted its focus to the tumor microenvironment of SCLC [12,13]. Results have shown that the reason for immunotherapy resistance despite strong immune infiltration could be the overexpression of immunosuppressive molecules, such as annexin A1 (ANXA1) [13].

Annexin A1 belongs to the annexin superfamily of calcium-dependent phospholipid-binding proteins and has several physiological and pathological roles, among which its anti-inflammatory, glucocorticoid-like effect is the best characterized [14]. Regarding cancer pathology, ANXA1 has controversial roles. Depending on the tissue and cancer type, ANXA1 has been found upregulated or downregulated, and it is described as a tumor promoter or a tumor suppressor. The knockdown of originally high ANXA1 expression has been assessed previously in cancer cell lines, showing suppressive effects on the viability and proliferation of glioma cells [15] and squamous cell lung cancer cells [16]. A recent study showed the direct inhibitory effect of an anti-ANXA1 antibody on the growth of breast, pancreatic, ovarian, and colorectal cancer cell lines [17]. In small cell lung cancer, ANXA1 has not been comprehensively investigated so far, but its upregulation has been identified as a negative, tumor-promoting factor, such as in association with SCLC bone [18] and brain [19] metastases. It is also necessary to investigate the potential direct involvement of ANXA1 in intratumoral subtype heterogeneity, NE to non-NE transition, and the plasticity of SCLC.

In this study, we aimed to comprehensively characterize the roles of annexin A1 in SCLC. To identify the molecule’s association with immune-infiltrated but immunosuppressed SCLC, we applied predictive algorithms using RNA-seq data from a real-word patient cohort and assessed the *in situ* expression of the protein in the tumor microenvironment in SCLC samples. We also aimed to reveal the functional role of ANXA1, by evaluating its direct effect on cancer cell growth and its effects on chemosensitivity and gene expression changes in mesenchymal markers, metabolic markers, dominant molecular subtype-specific transcriptional drivers, and immune-relevant molecules using in vitro short hairpin (sh) RNA-based gene silencing on non-NE SCLC cell lines. This is the first comprehensive characterization of annexin A1 in SCLC.

## 2. Materials and Methods

### 2.1. Predictive Algorithms and RNA-Seq Dataset

Whole-genome RNA-sequencing (WGS) data from the “UCologne2015” cohort, hosted within the comprehensive genomic database cBioPortal [20], was used to implement predictive algorithms and differential expression (DE), pathway, and survival analyses. We included *n* = 81 patients with available bulk WGS RNA-seq data using their CLR-normalized FPKM values. The samples were split based on the median expression of the ANXA1 gene and stratified into ANXA1-high and -low groups. We predicted and estimated infiltrating immune and stromal cells in tumor tissue via the immune, stroma, and purity scores of the ESTIMATE algorithm [21] provided by the hacksig (v0.1.2.) R package. Additionally, the expression data were deconvolved to obtain the proportions of immune, stromal, endothelial, and cancer cells using the EPIC algorithm [22] (R implementation v1.1). For both applications, only transcripts of the MANE Select (v1.2 matched annotation of NCBI and EMBL-EBI (https://www.ncbi.nlm.nih.gov/refseq/MANE/)) set were used, which consisted of one biologically representative transcript per protein-coding locus. MANE Select transcripts are identified using computational methods, complemented by manual review and discussion [23].

### 2.2. Differential Expression and Pathway Analyses

To determine differentially expressed genes (DEGs) in the ANXA1-high vs. -low subsets, we performed DEG analysis on the “UCologne2015” WGS dataset, starting with the following filtering steps. First, we excluded all genes not contributing a minimum of 0.001% to the total abundance and not present with an abundance greater than 0 in at least 20% of patients (~12,000 genes remained). Next, we used two well-established gene panels, the HTGEdge Oncological biomarker panel (*n* = 2549) and ImmuneDB’s Immunome panel (*n* = 844), to further narrow down the gene set and include only relevant genes. After the second filtering step, *n* = 1789 genes remained from the HTGEdge panel and *n* = 484 genes remained from the Immunome panel for DE analysis, conducted using DESeq2 (version 3.20). Gene expression counts were normalized, and dispersion estimates were obtained before fitting a negative binomial generalized linear model. Significantly differentially expressed genes were identified using Wald tests, with false discovery rate correction performed using the Benjamini–Hochberg method. DEGs were visualized on a Volcano plot by plotting the log10 (*p*-value) against the log2 fold change for each gene. Pathway analyses were performed separately using genes showing significant differential expression in ANXA1-high and ANXA1-low samples (threshold: adjusted *p*-value < 0.05; LogFC > [1.5]). Thus, we included *n* = 351 genes from the DEGs derived from the HTGEdge panel and *n* = 120 genes from the DEGs derived from ImmuneDB’s Immunome panel. For pathway analyses, the Reactome database was utilized, and data were generated with the WebGestalt software v2024 package. Over-representation analysis (ORA) was used to determine the enrichment ratios and false discovery rate (FDR) for every pathway. The affinity propagation method was used to reduce the redundancy of the gene sets in the enrichment.

### 2.3. Tumor Samples, Immunohistochemistry, and Immunofluorescence

Formalin-fixed paraffin-embedded (FFPE) tumor samples were kindly provided by the Department of Pathology and Experimental Cancer Research, Semmelweis University, Budapest, Hungary, from *n* = 9 early-stage SCLC patients. Patient data were completely pseudonymized, with no further clinical data used in this study due to the purely qualitative nature of immunostaining in tumor samples. Immunohistochemistry (IHC) and immunofluorescence were carried out as previously described [13]. Briefly, five-micron-thick sections were cut and stained using mouse monoclonal antibodies for rabbit monoclonal antibodies for annexin A1 (#32934, Cell Signaling) diluted 1:1000 during IHC staining. Endogenous peroxidase activity was quenched using 2% H_2_O_2_ solution. Tissue was permeabilized with 0.5% Triton X-100. The primary antibody-binding spots were visualized using the ImmPRESS© HRP Universal Polymer Kit (peroxidase, horse anti-mouse/rabbit IgG, vector) and exposed to epitope retrieval (low-pH citrate solution) for 20 min prior. Counterstaining was performed with hematoxylin. For double IF staining, primary antibodies for CD45 (#40763, abcam), CD68 (#303565, abcam), CD3 (#5690, abcam), CD19 (#90176, Cell Signaling), vimentin (#MA5-11883, Invitrogen), and ANXA1 (#32934, Cell Signaling) and fluorescent antibodies, namely Alexa IgG anti-rabbit A488 and IgG anti-mouse A594, were used as secondaries (Invitrogen). The TrueVIEW© Autofluorescence Quenching Kit (vector) was used to eliminate autofluorescence on FFPE samples. The visualization of cell nuclei was carried out with 4′,6-diamidino-2-phenylindole (DAPI). Protein expression detection was optimized in human tonsil and lung adenocarcinoma tissue as a positive control. IHC and IF staining was imaged using a Zeiss LSM 780 laser-scanning confocal microscope (Zeiss, Oberkochen) with 10× and 20× magnification objectives.

### 2.4. Cell Lines, Transfection, and Culture Conditions

Three SCLC cell lines, NCI-H841 (RRID: CVCL_1595), SW1271 (RRID: CVCL_1716), and NCI-H1048 (RRID: CVCL_1453), were purchased from the American Type Culture Collection (Manassas, VA, USA). All experiments were performed with authenticated, Mycoplasma-free cells. An annexin A1 silencing lentiviral shRNA vector (shANXA1; MISSION^®^ TRC2 pLKO.1-puro-CMV-tGFP TRCN0000296381, reference sequence NM_000700 human 3UTR, target sequence: 5′-CTATGATCAGAAGACTTTAAT-3′, Sigma-Aldrich) with a control short hairpin RNA (SHC202V MISSION^®^ TRC2 pLKO.5-puro non-mammalian shRNA control transduction particles) was purchased from Sigma-Aldrich. Transfection was performed according to the manufacturer’s protocol, with 8 µg/mL hexadimethrine bromide (Polybrene, #TR-1003, Sigma-Aldrich). Cells were cultured in low-glucose Dulbecco′s modified Eagle′s medium (#D6046, Sigma-Aldrich), supplemented with 10% fetal bovine serum (#TMS-016, Sigma-Aldrich) and 1% penicillin–streptomycin (#30-004-CI, Corning) in a humid, 37 °C atmosphere with 5% CO_2_. As a selection agent, 10 µg/mL puromycin (#ant-pr-1, Invivogen) was added to the culture medium of transfected cells. Native, shCTRL, and shANXA1 cells were cultured in parallel for experiments for a maximum of two passages. The experimental protocol and results for native cells are shown in Supplementary Figures S1–S5.

### 2.5. Trypan Blue Exclusion Growth Rate Assay

Transfected shCTRL and shANXA1 cells were seeded in 24-well plates (#130186, Thermo Fisher Scientific) in technical triplicates. The seeding densities were 50,000 cells/well for SW1271, 25,000 cells/well for H841, and 50,000 cells/well for H1048. Cells were detached with TrypLE™ Express Enzyme (1X) and no phenol red (#12604021, Gibco), collected with centrifugation at 200 rcf for 5 min, and counted in a hemocytometer at 48 h, 72 h, 96 h, and 120 h, using 0.4% trypan blue solution at a 1:1 ratio (#SV30084.01, Cytiva HyClone™).

### 2.6. Assessment of Chemosensitivity and Growth Rate with Presto Blue Viability Assay^TM^

Cells were seeded into five 96-well plates (#353377, Corning) at a seeding density of 5000 cells/well, with randomized plate layouts for each experiment, with technical triplicates. After seeding, cells were incubated overnight to attach, and three plates for 72 h, 96 h, and 120 h measurements were treated with etoposide (#12092, Cayman Europe) or cisplatin (#S1166, Avidin Ltd.) the next day at the following concentrations: 5 µM, 10 µM, 20 µM. Two plates were not treated for 0 h and 48 h measurements for the assessment of the 120 h growth rate in parallel (shown in Supplementary Figure S5). Non-treated controls were included in each plate and served as negative controls and references for the normalization of the results. As a positive control, 0.1% staurosporine (#S6942, Sigma-Aldrich) was used on shANXA1 cells. The vehicle of etoposide was 0.1% dimethyl sulfoxide (#D2650, Sigma-Aldrich), which was included as a 0.1% vehicle control in the etoposide chemosensitivity experiments. Cisplatin was dissolved in the culture medium. The plates for the 96 h and 120 h experiments were re-treated at 72 h. Resazurin–resorufin reduction-induced fluorescence was measured with the Presto Blue Viability Assay^TM^ (#A13261, Invitrogen) once on each of the five plates, at treatment and at 48 h, 72 h, 96 h, and 120 h after treatment, according to the manufacturer’s protocol. This was achieved using a Varioskan Lux multimode microplate reader (Thermo Fisher Scientific, Waltham, MA, USA) at room temperature, using a 560 nm excitation wavelength and 590 nm emission wavelength. The results are shown as relative fluorescence intensity units (RFU), normalized to our own non-treated controls. 

### 2.7. Western Blot Analysis

Western blot experiments were carried out as previously described [24]. The following antibodies were used in the experiments: annexin A1 (D5V2T) XP^®^ rabbit mAb (#32934, Cell Signaling), citrate synthase rabbit mAb (#ab129095, AbCam), LDHA (C45B) rabbit mAb (#3582, Cell Signaling), vimentin rabbit pAb (#bs-0756, Bioss), ß-catenin (D10A8) XP^®^ rabbit mAb (#8480, Cell Signaling), YAP1 rabbit pAb (#PA1-46189, Invitrogen), POU2F3 rabbit pAb (#av32537, Sigma Aldrich), CD-47 rabbit pAb (#ab175388 Abcam), PD-L1 E1L3N^®^ XP^®^ rabbit mAb (#13684), β-actin (13E5) rabbit mAb (#4970, Cell Signaling), GAPDH (D16H11) XP^®^ rabbit mAb (#5174, Cell Signaling). For the applied dilutions, see Supplementary Table ST1.

Cells were detached with TrypLE Express solution and centrifuged at 130 rcf for 5 min, and the pellet was resuspended in lysis buffer containing 1X radio immunoprecipitation assay (RIPA) buffer (#9806, Cell Signaling) and 1X HaltTM protease inhibitor cocktail (#78429, Thermo Fisher Scientific) on ice. Cell lysates were homogenized by sonication for 3 × 15 s and centrifuged twice at 10,000 rcf at 4 °C for 15 min to purify the protein extracts. Protein concentrations were measured with the bicinchoninic acid (BCA) assay (PierceTM BCA Protein Assay Kit, #23225, Thermo Fisher Scientific). An equal protein concentration of samples was reached by mixing the protein extracts with lysis buffer and a 1/4 volume of Laemmli buffer containing β-mercaptoethanol (#16010747, Bio-Rad). Samples were loaded on 26-well 4–20% Criterion™ TGX™ Precast Midi Protein Gels (#5671095, Bio-Rad), and electrophoresis was performed. Samples were then transferred onto polyvinylidene difluoride (PVDF) membranes with the Trans-Blot Turbo RTA Midi 0.2 µm PVDF Transfer Kit (#1704273, Bio-Rad). Membranes were blocked in 5% bovine serum albumin (#9998, Cell Signaling) in 0.05% Tris-buffered saline (TBS-T) (#10376743, Fisher Bioreagents) containing 0.05% Tween-80 (#P4780, Sigma Aldrich) for 2 h at room temperature and then probed with primary antibodies overnight at 4 °C. Membranes were washed three times with TBS-T, and anti-rabbit IgG HRP-linked antibody (#7074 Cell Signaling) was added for two-hour-long incubation at room temperature. The membranes were re-washed in TBS-T three times. Chemiluminescent signals were visualized using an enhanced chemiluminescence kit (#1705061, Bio-Rad) by a Chemidoc XRS+ or MP+ imaging system. Densitometry analysis was performed with the Image Lab™ 6.0 software (Bio-Rad). Results are shown as relative expression compared to ß-actin or GAPDH loading controls and displayed on graphs normalized to the native group on the annexin A1 Western blot and to the shCTRL group in all other experiments. The uncropped western blot images are available in the Supplementary Materials (Figures S6–S14).

### 2.8. Statistical Analysis

**Figure 1:**
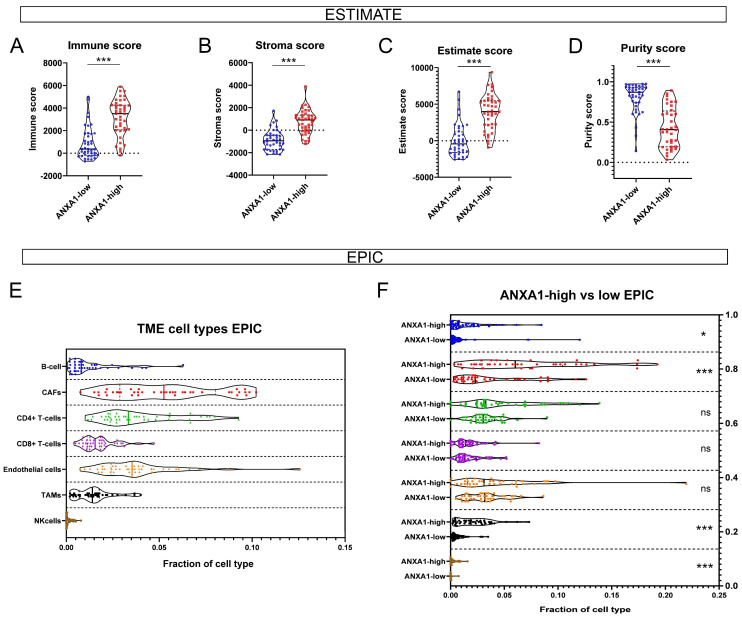
Predictive characterization of the SCLC TME according to ANXA1 expression. ESTIMATE algorithm revealed significantly higher immune (*p* < 0.001) (**A**) and stroma scores (*p* < 0.001) (**B**) in ANXA1-high tumors and indicated higher estimate (*p* < 0.001) (**C**) and lower purity scores (*p* < 0.001) (**D**). EPIC algorithm shows the distribution of the main TME cell types in SCLC tumors derived from bulk RNA-seq data (**E**). ANXA1-high tumors were shown to harbor significantly more B-cells (*p* = 0.027), CAFs (*p* < 0.001), TAMs (*p* < 0.001), and NK cells (*p* < 0.001) compared to ANXA1-low tumors (**F**). Data are displayed on violin charts, where the median (solid line) and upper and lower quartiles (dashed lines) are indicated. Wilcoxon’s rank sum test was performed for pairwise comparisons, where no data showed a normal distribution according to the Shapiro–Wilk test. ns: non-significant, *: *p* < 0.05, ***: *p* < 0.001.

Statistical analysis was performed with GraphPad Prism 8.0.0 (GraphPad Software, Inc., Boston, MA USA). Growth rate assays were evaluated using one-way ANOVA, followed by Tukey’s post hoc test. For the chemosensitivity assays, two-way ANOVA followed by Tukey’s post hoc test was used. The comparison of the shCTRL and shANXA1 Western blots was performed with an unpaired t-test or the Mann–Whitney test. Data were assessed with the Shapiro–Wilk test for normality. Experiments including the native, non-transfected group were evaluated by one-way ANOVA followed by Tukey’s multiple comparisons test (see annexin A1 Western blot in Figure 1B and all results with native cells in Supplementary Figures S2–S5). Group sizes were *n* = 6–8; all data are expressed as the mean ± standard error of the mean (SEM). A *p* value less than 0.05 was considered statistically significant. Survival analysis was performed with Kaplan–Meier curves and a comparison of the survival curves with the log-rank test. For multivariate Cox-proportional hazard regression, the analysis was two-sided, with a significance threshold of = 0.05. The predictive value of ANXA1 expression was tested with the confounders’ gender and tumor stage (early stage (I-II) vs. advanced stage (III-IV)). Harrel’s C-index was calculated to assess the quality of fit of our multivariate model, which was above 0.7 (fair) in all analyses.

## 3. Results

### 3.1. High ANXA1 Expression Is Associated with Increased Immune Infiltrates and Stromality

In our previous study, we showed that increased ANXA1 expression was prominent in immune-infiltrated and neuroendocrine (NE)-low SCLC [13]. Therefore, we aimed to investigate the TME characteristics of tumors using the open-access bulk RNA-seq data of *n* = 81 SCLC patients. We stratified the patients as ANXA1-high and ANXA1-low based on their median normalized RNA expression to estimate the immune cell infiltration and cell composition of tumors with the ESTIMATE [21] and EPIC [22] predictive algorithms.

The predictive algorithm ESTIMATE shows that ANXA1-high tumors exhibit significantly higher immune and stroma scores compared to ANXA1-low tumors (Figure 1A,B). The estimate score is derived from the sum of the immune and stroma scores, whereas the purity score acts as the inverse of the estimate score, explaining the relative proportion of tumor cells to non-tumor cells (stroma, immune cells, endothelium, etc.). Therefore, ANXA1-tumors possessed a significantly higher estimate score and a significantly lower purity score compared to ANXA1-low tumors (Figure 1C,D). EPIC is a more sophisticated algorithm that is able to distinguish fractions of different TME cell types (Figure 1E). It shows that ANXA1-high tumors harbor significantly more B-cells, cancer-associated fibroblasts (CAFs), tumor-associated macrophages (TAMs), and natural killer (NK) cells compared to ANXA1-low tumors (Figure 1F). These results are in line with the higher stromality and immune infiltrates predicted by ESTIMATE.

### 3.2. ANXA1-High Tumors Differentially Express Immune-Related Genes and Pathways of Active Immunosuppression

**Figure 2:**
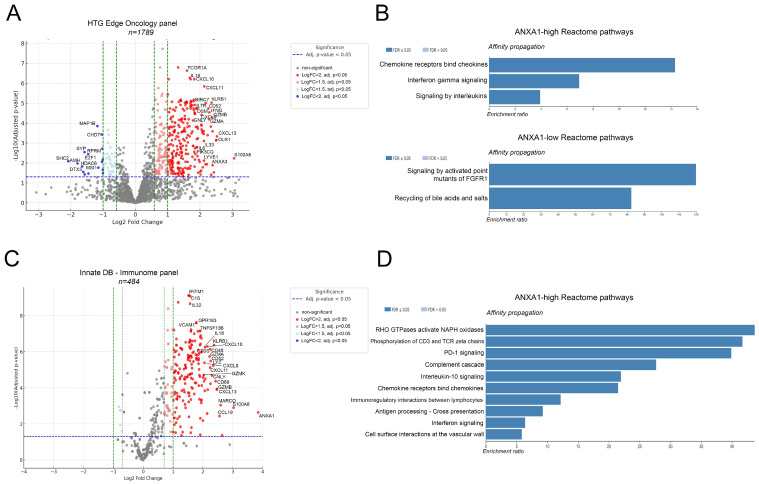
Differential expression and pathway analysis in the context of ANXA1 expression. Differentially expressed genes (DEGs) in ANXA1-high and ANXA1-low tumor subsets were analyzed using the HTG EdgeSeq oncological biomarker panel. *n* = 230 genes showed significantly increased expression in ANXA1-high tumors, while *n* = 16 genes were significantly overexpressed in ANXA1-low tumors (adjusted *p*-value < 0.05; LogFC > 2) (**A**). Pathway analysis using the Reactome database shows significant enrichment ratios (FDR ≤ 0.05) in blue for *n* = 3 pathways in ANXA1-high and *n* = 2 pathways in ANXA1-low tumors (**B**). Using ImmunDB’s Immunome panel, *n* = 117 genes were significant in ANXA1-high tumors and none in ANXA1-low tumors (**C**). Pathway analysis performed as described previously shows the 10 most enriched pathways (FDR ≤ 0.05) in ANXA1-high tumors. The affinity propagation method was used to reduce the redundancy of the gene sets in the enrichment (**D**).

To assess differentially expressed genes (DEGs) in the ANXA1-high and -low tumor subsets, we performed DEG analysis for two focused gene sets of oncological and onco-immunological importance. First, we used genes included in the HTG EdgeSeq oncological biomarker panel to identify DEGs in different patient groups. *n* = 230 genes showed strong significance regarding increased expression in the ANXA1-high group and *n* = 16 genes did so in the ANXA1-low group according to our criteria (adjusted *p*-value < 0.05; LogFC > [2]; Figure 2A), including FCGR1A, IL18, CXCL10, CXCL11, IL7R, CD52, CXCL13, KLRB1, OLR1, IFNG, GZMA, S100A8, and IL6 in ANXA1-high and SHC2, SYP, DTX3, CHD7, and MAP1B in ANXA1-low (Figure 2A). To extract meaningful biological information from the DEGs, we included all DEGs with an adjusted *p*-value < 0.05 and LogFC > [1.5] in our pathway analysis performed on the Reactome database. ANXA1-high tumors were enriched in immunology-related pathways such as chemokine receptor–ligand interactions and interferon gamma and interleukin signaling, whereas ANXA1-low tumors were enriched in pathways of cancer cell biology, such as the signaling of activated point mutants of FGFR1 and recycling of bile acids and salts (Figure 2B).

We also performed a DEG analysis on a more focused gene subset, ImmunDB’s Immunome panel, where only unequivocally immune system-related genes remained in our data pool. This time, *n* = 117 genes showed strong significance in differential expression, all of them in the ANXA1-high group, with many being similar to the previous analysis and with some newly identified, including GPR183, VCAM1, CD69, CD48, LYZ, and GNLY (Figure 2C). The pathway analysis on the Reactome database showed more specific pathways that were significantly enriched in ANXA1-high tumors, many of them associated with active immunosuppression: the phosphorylation of CD3 and TCR zeta chains, PD-1 signaling, the complement cascade, or interleukin-10 signaling (Figure 2D).

### 3.3. ANXA1-High Patients Exhibit Significantly Decreased Overall Survival

**Figure 3:**
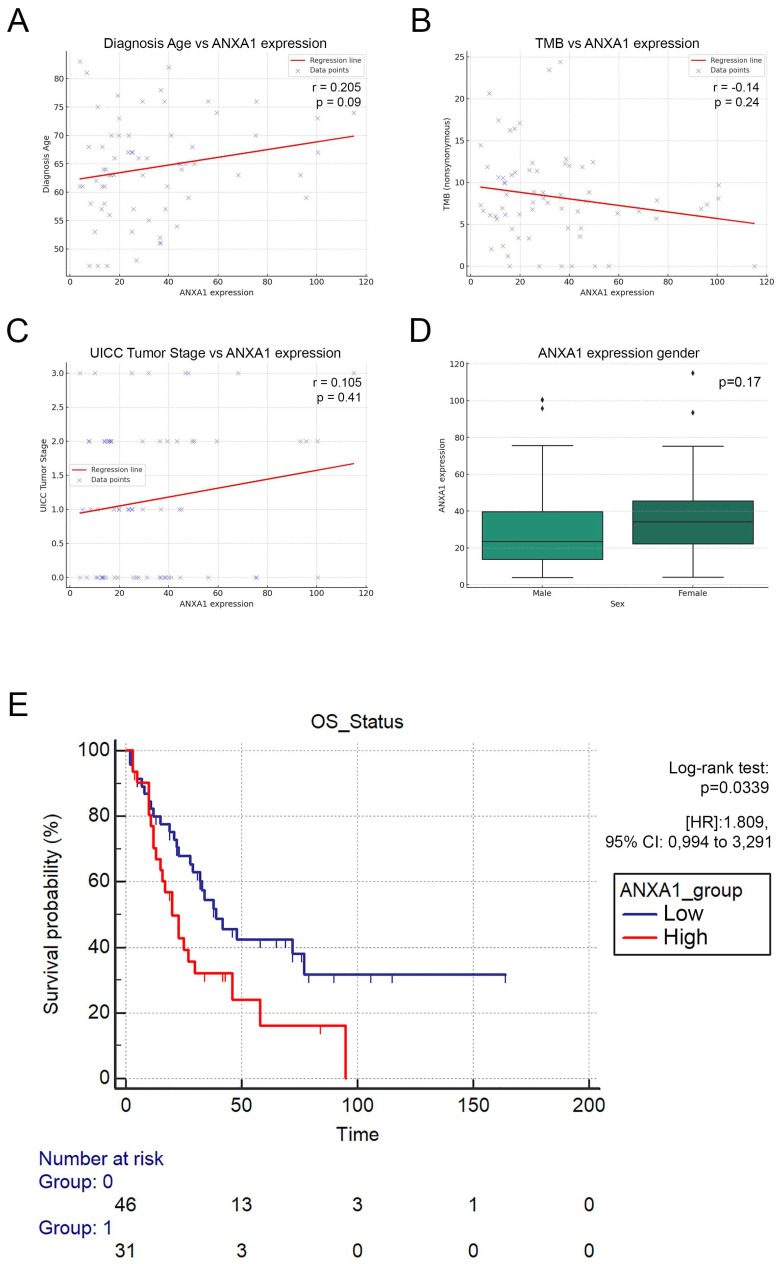
Clinical characteristics of ANXA1 expression. Clinical parameters and overall survival (OS) were evaluated in the SCLC cohort according to ANXA1 expression. Spearman correlation and pairwise comparisons showed no significant associations between ANXA1 expression and diagnosis age (**A**), TMB (**B**), tumor stage (**C**), or gender (**D**). Kaplan–Meier [25] analysis indicated significantly decreased OS in ANXA1-high patients (HR: 1.809, 95% CI: 0.994 to 3.291, *p* = 0.0339, (**E**). Log-rank test was used to establish significance in KM analysis. Wilcoxon rank sum test was performed for pairwise comparison according to gender. Spearman’s correlation analysis was performed to assess coefficients (rs) and *p*-values (*p*) when comparing ANXA1 expression with diagnosis age, TMB, and tumor stage.

Next, we aimed to evaluate the clinical parameters and overall survival (OS) in the publicly available SCLC cohort according to ANXA1 expression. We performed a Spearman correlation analysis and pairwise comparison to reveal any connections between the diagnosis age, TMB, tumor stage, gender, and ANXA1 tumor expression, where none of the clinical parameters showed significant associations with the latter (Figure 3A–D). The Kaplan–Meier analysis, however, showed that ANXA1-high patients showed significantly decreased OS compared to ANXA1-low patients ([HR]: 1.809, 95% CI: 0.994 to 3.291, *p* = 0.0339, Figure 3E). Furthermore, when including the two most relevant confounders and ANXA1 expression in multivariate Cox regression, ANXA1 expression showed significant prognostic power, similar to that of gender ([HR]: 2.046, 95% CI: 1.1471 to 3.6504, *p* = 0.0158, Table 1).

### 3.4. ANXA1 Protein Is Expressed on Tumor Cells and Macrophages in SCLC Tumor Samples

**Figure 4:**
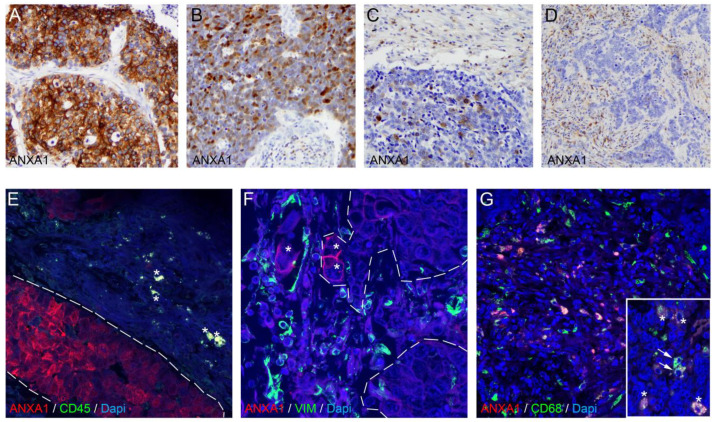
Tissue expression of ANXA1 protein. ANXA1 protein expression was evaluated using IHC and double IF staining on resected tumors from 9 early-stage SCLC patients. ANXA1 expression was observed in the tumor cells of 2 patients, with scattered stromal cell positivity (**A**,**B**). Two additional patients showed ANXA1 expression in the stroma and in non-tumoral cells within tumor nests (**C**,**D**). Double IF staining revealed ANXA1 co-expression with CD45 on immune cells (**E**, asterisks), whereas cancer cells of tumor nests showed positivity only for ANXA1. ANXA1+ tumor cells (**F**, asterisks) do not colocalize with vimentin+ CAFs in the stroma (**F**), whereas ANXA1 shows expression on multiple CD68+ TAMs both in the stroma (**G**) and in tumor nests (**G**, asterisks in inset). In contrast, some CD68+ TAMs show no co-expression of ANXA1 (**G**, arrows in inset).

We evaluated the expression of the ANXA1 protein with IHC and double IF staining on the resected tumors of *n* = 9 early-stage SCLC patients to perform a qualitative analysis of *in situ* ANXA1 expression. Two patients showed the presence of the protein in tumor cells, where scattered cells in the stroma also showed positivity (Figure 4A,B). Furthermore, we found two more patients with ANXA1 expression only in the stroma and on non-tumoral cells inside tumor nests (Figure 4C,D). *n* = 5 patients showed no signs of ANXA1 protein expression either in tumor or in stromal cells. To identify those cells that expressed ANXA1 in the stroma, we performed double IF staining for pan-leukocyte marker CD45, CAF marker vimentin, pan-macrophage marker CD68, T-cell marker CD3, and B-cell marker CD19. We found that multiple cells with an amoeboid and ramified morphology co-expressed CD45 and ANXA1 in the tumor stroma, showing the protein’s presence on immune cells (Figure 4E). In contrast, vimentin expression on stromal cells did not show colocalization with ANXA1 expression (Figure 4F). CD68, however, was co-expressed with ANXA1 throughout on the majority of TAMs, both in the stroma and in tumor nests (Figure 4G). We found no ANXA1 co-expression with CD3 or CD19 in any of the tumors, indicating that ANXA1 is expressed on tumor cells and TAMs in the TME.

### 3.5. Annexin A1 Expression Is Stably Silenced in shANXA1 Transfected Cells

**Figure 5:**
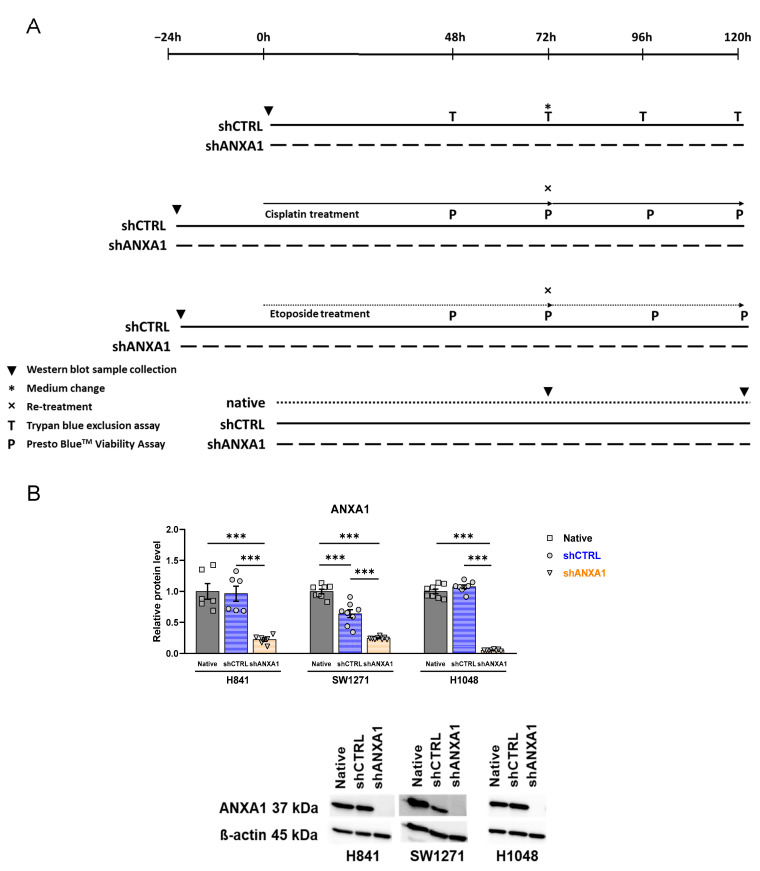
Experimental protocol and annexin A1 silencing. Groups and experimental protocol (**A**). Western blot evaluation of ANXA1 relative protein levels of native, shCTRL, and shANXA1 cells (**B**). One-way ANOVA, Tukey’s post hoc test, *n* = 6–8; *** *p* < 0.001. Data are expressed as mean ± SEM.

To comprehensively characterize the direct roles of ANXA1 in SCLC, we assessed *in vitro* endpoints of the malignant phenotype in cancer cell lines. The experimental design of the *in vitro* studies is shown in Figure 5A. The three SCLC cell lines H841, SW1271, and H1048 expressed annexin A1 at higher levels in comparison with 25 other SCLC cell lines, with H1048 cells showing the strongest expression, as reported previously [13]. Western blot analysis showed the stable silencing of ANXA1 expression in shANXA1 cells in all three cell lines (Figure 5B). In H841 and H1048 cells, transfection with the shCTRL vector did not change ANXA1 expression significantly, while, in SW1271 cells, significantly decreased—but, as compared with shANXA1 cells, significantly maintained—ANXA1 expression was observed (Figure 5B).

### 3.6. Effects of ANXA1 Silencing on Growth Rate

**Figure 6:**
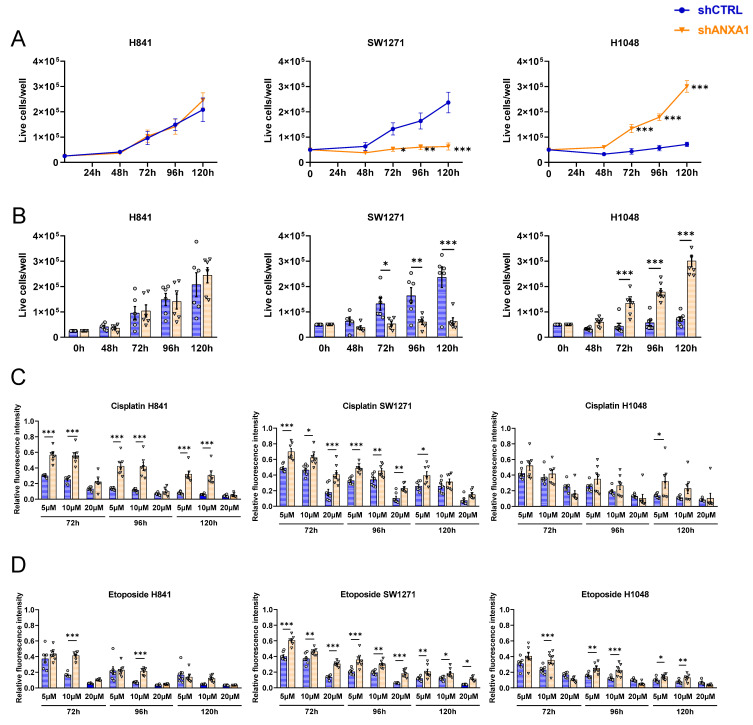
Growth rate and chemosensitivity assays. Growth rate assay shown as 120-h-long growth curve (**A**) and as individual data at time points (**B**). Trypan blue exclusion assay, *n* = 6, one-way ANOVA, Tukey’s post hoc test, * *p* < 0.05, ** *p* < 0.01, *** *p* < 0.001. Data are displayed as mean ± SEM. Cisplatin (**C**) and etoposide (**D**) chemosensitivity assay. Presto Blue^TM^ Viability Assay, *n* = 6–7; two-way ANOVA, Tukey’s post hoc test, * *p* < 0.05, ** *p* < 0.01, *** *p* < 0.001. Data are displayed as mean ± SEM.

We performed the trypan blue exclusion growth rate assay for 120 h. Annexin A1 silencing affected the growth rates of all three cell lines in different ways. In H841 cells, shCTRL and shANXA1 cells had similar proliferation rates, with no tendencies to reach statistical significance (Figure 6A,B, first panel). In SW1271 cells, shANXA1 cells grew significantly more slowly than shCTRL cells, and the difference became more significant after a longer culturing period (Figure 6A,B, second panel). Interestingly, in H1048 cells, shANXA1 cells proliferated significantly faster than shCTRL cells, and this difference was marked from 72 h until the end of the experiments, with the greatest difference observed at 120 h (Figure 6A,B, third panel). We present the results with growth curves (Figure 6A) and on bar graphs, showing individual data points (Figure 6B).

### 3.7. Effects of ANXA1 Silencing on Cisplatin and Etoposide Chemosensitivity

First-line treatment agents in SCLC chemotherapy, namely cisplatin and etoposide, were used on the cell lines to assess whether their chemosensitivity was affected by ANXA1 silencing *in vitro*. We applied a 120-h-long incubation period based on the growth rate results. Annexin A1 silencing, in general, decreased the chemosensitivity to both agents in all three cell lines (Figure 6C,D).

Despite the similar growth rates of H841 shCTRL and shANXA1 cells, H841 shANXA1 cells were significantly less sensitive to cisplatin-induced cell death in the 5 µM and 10 µM treatments, at 72 h, 96 h, and 120 h (Figure 6C, first panel). Treatment with 20 µM cisplatin resulted in similarly diminished viability in both the shCTRL and shANXA1 groups at each time point in H841 cells (Figure 6C, first panel). The etoposide treatment of H841 cells resulted in significantly decreased chemosensitivity in shANXA1 cells at 10 µM in the 72 h and 96 h measurements, and the same tendency was seen at 10 µM at 120 h (Figure 6D, first panel). The lowest, i.e., 5 µM, and the highest, i.e., 20 µM, treatments did not show significant changes in viability between H841 shCTRL and shANXA1 cells at either time point (Figure 6D, first panel). SW1271 shANXA1 cells showed markedly higher viability at each time point and each concentration of both cisplatin and etoposide, reaching statistical significance at each condition, except in the 10 µM and 20 µM cisplatin treatments at 120 h, but the tendency was clear in these cases as well (Figure 6C,D, second panel). In H1048 cells, the 5 µM and 10 µM treatments resulted in the higher viability of shANXA1 cells with cisplatin and etoposide as well, at each time point. Due to the standard deviation of the data, only tendencies were seen in the cisplatin experiments (Figure 6C, third panel). With etoposide treatment, at the 5 µM concentration at 72 h and the 5 µM and 10 µM concentrations at 96 h and 120 h, H1048 shANXA1 cells showed significantly higher viability (Figure 6D, third panel). Interestingly, the 20 µM treatments with both cisplatin and etoposide resulted in a tendency towards greater cell death in H1048 shANXA1 cells in response to cisplatin at 72 h and 96 h (Figure 6C, third panel) and to etoposide at each time point (Figure 6D, third panel).

### 3.8. Effects of ANXA1 Silencing on Expression of Metabolic and Mesenchymal Markers

**Figure 7:**
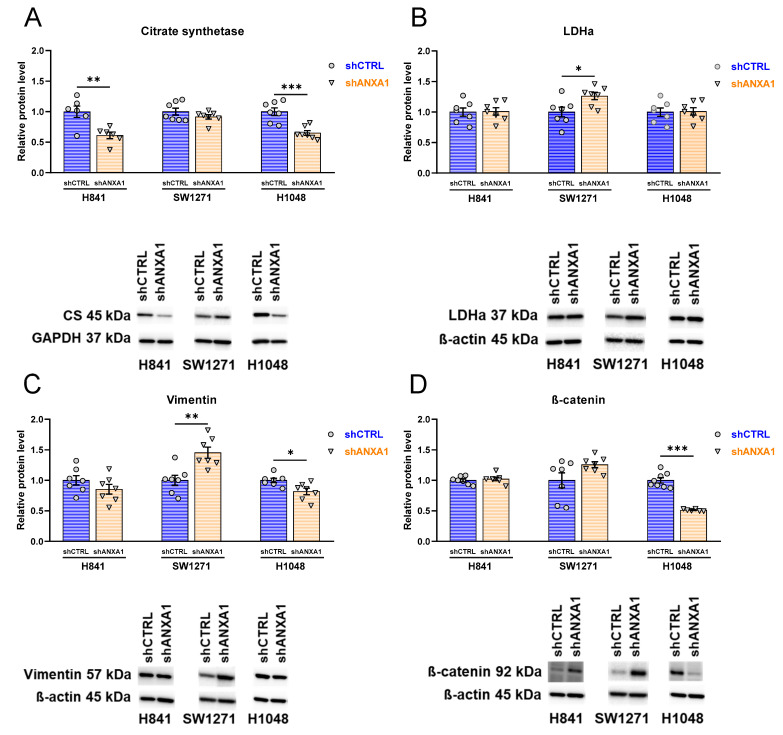
Western blot evaluation of metabolic and mesenchymal markers. Relative protein levels of citrate synthetase (**A**), lactate dehydrogenase a (LDHa) (**B**), vimentin (**C**), and ß-catenin (**D**)**.** Western blot, *n* = 6–7; normalized for shCTRL group of each cell line. Unpaired t-test, * *p* < 0.05, ** *p* < 0.01, *** *p* < 0.001. Data are displayed as mean ± SEM.

To assess metabolic changes in SCLC cell lines in response to ANXA1 silencing, we selected citrate synthase (CS) as a marker of oxidative metabolism and lactate dehydrogenase (LDH) as a marker of anaerobic glycolysis, with the latter being the preferred pathway for ATP production in cancer cells showing the Warburg phenomenon [26]. Citrate synthase expression was significantly decreased in H841 shANXA1 and H1048 shANXA1 cells as compared with shCTRL cells (Figure 7A). SW1271 shCTRL and shANXA1 cells did not show significantly changed citrate synthase expression, with only a slight decrease in shANXA1 cells (Figure 7A). LDH expression did not change in H841 shANXA1 and H1048 shANXA1 cells, while it was significantly increased in SW1271 shANXA1 cells (Figure 7B).

We assessed the expression of two mesenchymal markers, vimentin and ß-catenin, to evaluate the mesenchymal nature as a malignant feature of cancer cells. Vimentin expression was slightly decreased in H841 shANXA1 and H1048 shANXA1 cells, reaching statistical significance in H1048 shANXA1 cells as compared to H1048 shCTRL cells (Figure 7C). However, vimentin expression was significantly increased in SW1271 shANXA1 cells (Figure 7C). ß-catenin showed no changes in H841 cells in response to ANXA1 silencing and showed a tendency towards increased expression in SW1271 shANXA1 cells (Figure 7D). In H1048 cells, ß-catenin expression was significantly decreased in shANXA1 cells in comparison with shCTRL cells (Figure 7D). To conclude, mesenchymal marker expression did not change in H841 cells in response to ANXA1 silencing, and it slightly increased in SW1271 shANXA1 cells. However, the microscopic observation that the ANXA1-silenced H1048 cells were much more adherent (see photos in Supplementary Figure S1B) could hypothetically be attributed to decreased mesenchymal marker expression in H1048 shANXA1 cells, as shown with the Western blot.

### 3.9. Effects of ANXA1 Silencing on Expression of Dominant Transcriptional Drivers and Immune-Relevant Markers

**Figure 8:**
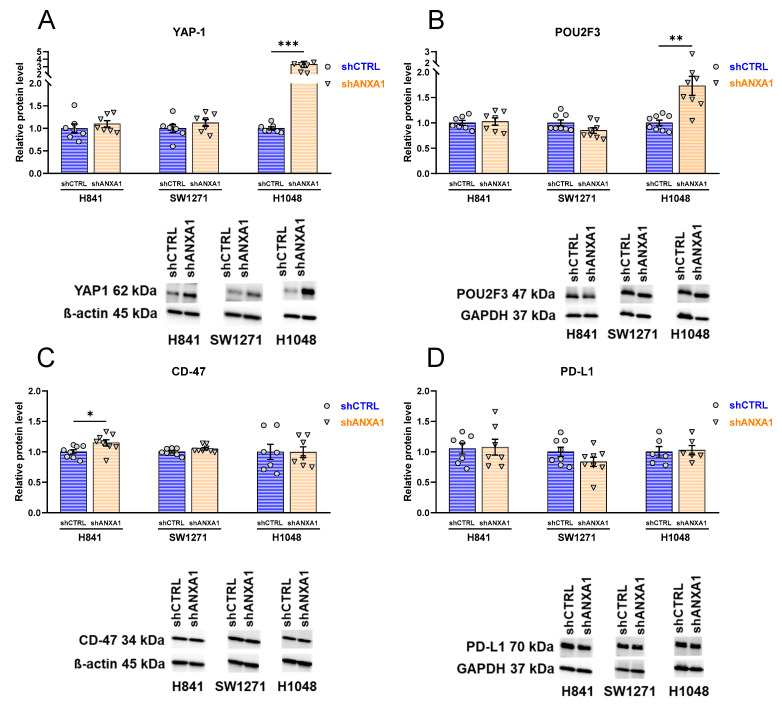
**Western blot evaluation of dominant transcriptional drivers and immunomodulatory markers.** Relative protein levels of YAP1 (**A**), POU2F3 (**B**), CD-47 (**C**), and PD-L1 (**D**). Western blot, *n* = 6–8; normalized for shCTRL group of each cell line. Unpaired t-test, * *p* < 0.05, ** *p* < 0.01, *** *p* < 0.001. Data are displayed as mean ± SEM.

Of the three SCLC cell lines, H841 and SW1271 have YAP1 as the dominant transcriptional driver, while H1048 has POU2F3, as described previously [13]. YAP1 expression showed a slight further increase in H841 shANXA1 and SW1271 shANXA cells and a three-fold increase in H1048 shANXA1 cells as compared with shCTRL cells (Figure 8A). POU2F3 expression did not change significantly in ANXA1-silenced H841 and SW1271 cells; however, a slight decrease was observed in SW1271 shANXA1 cells. In H1048 cells, POU2F3 expression became significantly higher in shANXA1 cells than shCTRL cells (Figure 8B).

The expression of a tumor immune tolerance induction marker, CD-47, significantly increased in H841 shANXA1 cells in comparison with H841 shCTRL cells (Figure 8C). However, no significant changes in CD-47 expression were observed in the SW1271 or H1048 cell lines due to ANXA1 silencing (Figure 8C). The expression of PD-L1, a target of several approved immunotherapy agents, was not significantly changed in the ANXA1-silenced groups in either cell line; however, a slight decrease was detected in SW1271 shANXA1 cells (Figure 8D).

## 4. Discussion

SCLC remains one of the most lethal malignancies, with limited advancements in therapeutic options. The lack of targeted treatments and its complex TME contribute to poor patient outcomes. ANXA1, known for its immunomodulatory properties, has emerged as a significant molecule potentially influencing tumor and metastasis progression [27], along with an association with an immunosuppressive microenvironment [28,29,30]. In this study, we performed the comprehensive characterization of ANXA1 in SCLC, exploring its role in the TME, cancer cell growth, chemosensitivity, and gene expression.

We utilized RNA-seq data from a real-world patient cohort to predict immune infiltration and stromal cell presence in tumors. By stromality, we refer to the stromal score described in the predictive algorithm ESTIMATE, assessing the level of stromal cells present in the tumor tissue [21]. Stromal cells are specialized connective tissue cells, including fibroblasts and mesenchymal stromal cells [31]. The results revealed that ANXA1-high tumors exhibited significantly higher immune and stromal scores compared to ANXA1-low tumors. These tumors also demonstrated the increased infiltration of CAFs and TAMs, characteristic of a more immunosuppressed TME. However, the predictive algorithm EPIC also revealed slightly increased B-cell numbers in ANXA1-high tumors, suggesting a multifaceted association between the molecule and the TME. Data indicate that ANXA1 is also involved in bidirectional crosstalk between macrophages and tumor cells, promoting M2-polarized TAM aggregation [32,33], and is involved in the activation of multiple immunosuppressive pathways, including PD-L1 signaling in glioblastoma [34], breast cancer [25,35], and other malignancies [30].

Our findings, together with existing evidence [13], suggest that TAMs are the dominant effectors driving ANXA1-mediated immunosuppression in SCLC through IL-10 and TGF-β secretion, while increased B-cells—potentially enriched in regulatory B-cells [36] —and CAFs [37] may further contribute to immune evasion by promoting Treg differentiation and remodeling the extracellular matrix, respectively.

We also observed that high ANXA1 expression was correlated with the upregulation of immune-related genes and immunosuppressive pathways, such as Rho GTPases activating NADPH oxidases, the phosphorylation of CD3 and TCR zeta chains, PD-1 signaling, the complement cascade, and interleukin-10 signaling. NADPH oxidases are membrane-associated enzymes using NADPH to produce superoxide (O2-) as a secondary messenger [38], interacting with the VEGF/VEGFR2 axis and enhancing angiogenesis [39]. When TCRs recognize antigens presented by MHC molecules on the surfaces of tumor cells or antigen-presenting cells, the CD3 and TCR zeta chains’ phosphorylation is essential during MHC-driven antigen presentation, initiating a cascade of downstream signaling leading to T-cell activation, proliferation, and differentiation, whose impairment can cause tumor immune evasion [40]. The overexpression of genes associated with immunosuppression, such as those involved in PD-1 and interleukin-10 signaling, underscores the potential for ANXA1 to drive mechanisms that allow tumors to escape immune surveillance [41,42]. TAMs and other TME elements can secrete cytokines like TGF-β and IL-10, which inhibit T-cell activation by interfering with the phosphorylation of CD3 and TCR zeta chains [40,42]. Addressing these suppressive influences is crucial for effective anti-tumor immunity and can be exploited in ANXA1-high tumors, where PD-1 blockade or anti-IL-10 therapies might be effective.

Immunostaining on tumor samples revealed ANXA1 protein expression in tumor cells and TAMs but not on CAFs or lymphocytes. ANXA1’s expression on tumor cells and macrophages, as demonstrated by IHC and double IF staining, suggests that it might facilitate a microenvironment conducive to tumor growth and resistant to immune attack [13,32,33]. Regarding its clinical context, ANXA1-high patients exhibited significantly decreased overall survival, with the Kaplan–Meier analysis showing an increase in the risk of death by approximately 80%, suggesting that ANXA1 is a negative prognostic marker, which was confirmed by the multivariate Cox regression. This finding is in line with other studies in glioma [43]; breast cancer, lung cancer, and melanoma [30,44]; pancreatic cancer [45]; and gastric cancer [46], where an association between higher ANXA1 expression and lower survival has been described previously, all suggesting the involvement of ANXA1 in increased invasiveness and metastatic potential. However, it contradicts results in ovarian cancer [47].

Furthermore, we assessed the direct impact of ANXA1 on cancer cell behavior through in vitro experiments using short hairpin RNA (shRNA)-based gene silencing in non-NE SCLC cell lines H1048 (POU2F3+), SW1271 (YAP1+), and H841 (YAP1+), which all show high levels of ANXA1 expression according to cell line data. Our findings indicate that ANXA1 silencing affects cell proliferation differently across cell lines, decreasing growth in SW1271 cells but increasing it in H1048 cells. Most recent research generally associates ANXA1 upregulation with increased proliferation through multiple cancers. Fang et al. found that ANXA1 silencing by a lentiviral siRNA vector suppressed the proliferation of A549 and H1299 cells [48]. Moreover, when investigating the connections between ANXA1 and SCLC bone metastases, Chen et al. found that, in the SBC-3 cell line, ANXA1 overexpression increased, while ANXA1 silencing decreased proliferation [18]. Similar results were reported in glioma [49]; papillary thyroid cancer [50]; and breast, pancreatic, ovarian, and colorectal ANXA1-expressing cancer cell lines [17]. However, the opposite has been reported in oral squamous cell carcinoma cell lines [51], and, in a 2021 study, no significant effect on proliferation was found in the H1299 and A549 NSCLC cell lines [52].

It is likely that the effects of ANXA1 expression are different even in different subtypes of the same cancer, through ANXA1 gene mutations, epigenetic changes, and post-translational modifications to the ANXA1 protein [53]. For instance, phosphorylation is needed for ANXA1 to localize on the cell surface [54]. The various roles of ANXA1 warrant further investigation of the precise mechanisms of ANXA1-mediated cellular activation. In breast cancer, ANXA1 has been described as both a tumor suppressor and an oncogene, with its expression levels depending on the molecular subtype. Estrogen receptor-positive breast cancers usually have low ANXA1 levels, while basal-like breast cancers show high ANXA1 expression, with both scenarios promoting tumor development in the corresponding tumor subtype [29].

The differential effects of ANXA1 silencing on cell growth across SCLC cell lines suggest that ANXA1 may interact with distinct cellular pathways in a context-dependent manner in this particular malignancy. This paradox could be explained by the intrinsic differences in the genetic/epigenetic landscapes or disparate NE subtypes of these cell lines. H1048 cells, which express high levels of ANXA1, may rely more heavily on ANXA1 for the regulation of pathways that limit proliferation; thus, its silencing removes a growth-suppressive constraint. Conversely, SW1271 cells might have alternative pathways that compensate for the loss of ANXA1, leading to a net reduction in proliferation.

Our chemosensitivity assays revealed that ANXA1 silencing generally decreased the sensitivity to cisplatin and etoposide across all cell lines, at concentrations that resembled patient plasma concentrations in vitro [55,56,57,58]. This result is not in line with most studies performed in other cancers, where ANXA1 upregulation was involved in mechanisms of chemoresistance, such as in triple-negative breast cancer cell line BT-549 [59], in oxaliplatin-resistant gastric cancer cells [60], and in the cisplatin-resistant A549 lung adenocarcinoma cell line [61]. However, decreased ANXA1 expression was also identified in adriamycin-resistant bladder cancer cell lines [62]. Reduced chemosensitivity may be linked to changes in cell cycle dynamics and apoptotic pathways influenced by ANXA1. The relative selectivity of chemotherapy is based on higher cell proliferation, and SW1271 shANXA1 cells had a significantly lower growth rate in comparison with shCTRL cells, suggesting no direct effect of ANXA1 silencing on SW1271 chemosensitivity but rather an escape from cytostatic effects due to decreased proliferation. The chemosensitivity results with H1048 cells cannot be clearly explained by the increased growth rate of H1048 shANXA1 cells. It is plausible that the markedly higher initial cell counts due to much higher proliferation rates resulted in a higher fluorescence intensity in the 5 µM and 10 µM treatments, but, in the 20 µM treatments, a chemosensitivity-increasing effect of ANXA1 silencing could be suggested. Of note, while high chemosensitivity can be an intrinsic property of some aggressive tumors due to their rapid cell division and specific genetic factors, this sensitivity can vary widely depending on the tumor type and a permissive TME. Thus, firm conclusions cannot be drawn from our findings in light of the disparate results of the cell line growth assays, which reflect the biological heterogeneity of SCLC.

The gene expression analysis after ANXA1 silencing highlighted significant changes in mesenchymal and metabolic markers, with variations observed across different cell lines. Altered expression levels of ANXA1, including both increases and decreases, have been linked to cancer progression and clinical outcomes [44,45,63,64]. To assess ANXA1’s role in tumor biology, we focused on two established hallmarks of cancer: the Warburg effect, which involves metabolic reprogramming, and the epithelial–mesenchymal transition (EMT). We used citrate synthase and lactate dehydrogenase as markers for the Warburg effect and vimentin and β-catenin as markers for EMT. These processes are strongly associated with tumor aggressiveness, making them important parameters when investigating the impact of ANXA1.

Notably, the mesenchymal marker vimentin decreased in H1048 cells while increasing in SW1271 cells. Changes in EMT were suspected based on the culturing characteristics of shCTRL and shANXA1 cells, especially in the H1048 cell line. ANXA1-silenced cells showed greater attachment to the culture dishes and required longer incubation times with TrypLE Express to detach, suggesting a more epithelial morphology and a phenotype with less invasive potential. In previous studies, ANXA1 mostly inhibited EMT and showed a negative correlation with vimentin expression. Our results were in line with this only in the SW1271 cell line; in H1048, we found decreased mesenchymal marker expression in ANXA1-silenced cells. Others showed that the knockdown of ANXA1 induced EMT in mammary epithelial cells [65] and in oral squamous carcinoma cells [51]. In lung cancer cell lines with non-small cell histologies (H1299 and A549), ANXA1 overexpression inhibited EMT, whereas ANXA1 silencing resulted in increased ß-catenin expression [52]. The variability in EMT and metabolic marker expression post-ANXA1 silencing also highlights the gene’s context-specific roles.

In a retrospective clinical study on over 300 limited- and extensive-stage patients, elevated LDH levels were associated with significantly lower survival in SCLC [66], whereas citrate synthase has cancer-type-specific roles: depending on the tumor’s metabolic profile, it can inhibit or promote tumor progression [67]. Concerning metabolic changes, our results suggest that ANXA1 silencing resulted in the significantly decreased oxidative metabolism of H841 and H1048 cells, while it slightly increased the anaerobic metabolism of SW1271 cells. In H841 and H1048 cells, citrate synthase expression decreased upon ANXA1 silencing, suggesting a shift away from oxidative metabolism towards a more glycolytic phenotype, which is commonly associated with the Warburg effect in cancer cells, promoting rapid growth, survival in adverse conditions, immune evasion, and invasiveness [68]. However, no clear anaerobic shift was observed, with only a minor increase in LDH activity in one of the cell lines. This might imply that tumor cells might be compensating for the reduced TCA cycle activity by relying on alternative metabolic pathways, like glutaminolysis [69]. Another possibility is that TCA activity may not be reduced considerably by the upregulation of enzymes that can feed intermediates into the TCA cycle downstream of citrate synthase. Moreover, these cells might maintain the redox balance and avoid lactate production by increasing the flux through the pentose phosphate pathway or other pathways that generate NADPH and help to manage oxidative stress. Additional metabolic assays, such as enzyme activity and oxygen consumption rate measurements, are needed to confirm these assumptions. Still, the ability of SCLC cells to maintain viability, or even growth and function, despite decreased citrate synthase activity and without elevating LDH, suggests a high degree of metabolic flexibility in SCLC.

The transcription factors YAP1 and POU2F3 can be dominant drivers in small cell lung cancer (SCLC), potentially defining emerging molecular subclasses that could influence treatment responses. Given the promising nature of this research area, we sought to explore whether changes in ANXA1 expression might affect these markers. In our study, YAP1 was the dominant transcriptional driver in the SW1271 and H841 cell lines, while POU2F3 had this role in the H1048 cell line. However, due to potential overlap and previously documented subtype shifts [11], we evaluated both markers across all cell lines. We also noted the differential expression of these markers: H1048 cells responded with the strong upregulation of non-NE marker YAP1 and POU2F3 expression upon ANXA1 silencing; however, there was no detectable change in the two other SCLC cell lines. These results suggest that, in H1048 cells, a downstream inverse dependence might exist between ANXA1 and non-NE subtype markers, but, due to its clonal and molecular heterogeneity, the universal nature of this effect in SCLC is not likely. YAP1 knockout resulted in the decreased proliferation of H69AR and SBC5 [70] and inhibited the growth of the H841 and SW1271 SCLC cell lines [71], which may imply that ANXA1 silencing caused increased proliferation in H1048 indirectly due to a surge in YAP1 expression. The reason for the lack of a proliferation boost in shANXA1 cells in the other SCLC cell lines might be their distinct molecular signaling patterns.

In a 2023 study by Xiao et al., ANXA1 was shown to upregulate PD-L1 expression in breast cancer, NSCLC, and melanoma cell lines [30]. While PD-L1 is a viable target and predictive marker in non-small cell lung cancer, its inhibitors have shown limited efficacy in small cell lung cancer, and the PD-L1-status is not predictive of the ICI response in SCLC, indicating that these tumors might be associated with different immune microenvironments [72]. Nevertheless, ANXA1 is also implicated in promoting tumor immune tolerance, and the PD-1/PD-L1 axis is the most well-characterized immune checkpoint. We investigated whether silencing ANXA1 would affect PD-L1 expression in any way. Although the predicted molecular weight of PD-L1 is 40–50 kDa, it was detected at 70 kDa in our Western blot experiments. This has been previously described using the same antibody on cellular lysates of human triple-negative breast cancer cell lines [73] and nuclear soluble components of human head and neck carcinoma cell lines [74]. This phenomenon was attributed to possible post-translational modifications. CD47, found on both healthy and malignant cells, regulates macrophage-mediated phagocytosis by sending a “don’t eat me” signal to the signal-regulatory protein alpha (SIRPα) receptor, being a popular target for clinical trials in cancer immunotherapy [75]. Increasing evidence demonstrates that blocking the interaction between CD47 and SIRPα can enhance cancer cell clearance by macrophages, which is particularly relevant in SCLC, where TAMs are the dominant cell type in the TME [13,76]. Despite the molecule’s relevance, we found no effect of ANXA1 silencing on CD47 expression in any of the tested cell lines, suggesting no signaling dependency between the two target proteins at the cellular level.

Our study has several limitations. First, the small number of patient samples included in the immunohistochemistry analysis did not allow us to validate the in silico and in vitro data reliably. However, obtaining patient samples is challenging due to the aggressive nature of the disease and current treatment protocols. Most patients are diagnosed at an advanced stage, where surgical interventions are rarely performed [77], limiting access to samples. As a result, we present an analysis of the nine cases that were available for this study. Gene silencing with shRNAs is limited by potential off-target effects, which cannot be completely eliminated. These effects can arise due to competition between shRNAs and endogenous miRNAs for processing and targeting, hypothetically exacerbated by sequence redundancy [78,79]. In our study, the shCTRL vector exhibited significant off-target effects in SW1271 cells, but ANXA1 expression was maintained, allowing us to use these cells as controls when comparing the effects of ANXA1 silencing versus retained expression for general characterization. However, controlling for off-target effects through in silico prediction or testing multiple shRNA sequences would be beneficial in future studies [80,81].

Future studies should include a broader range of cell lines and in vivo models to validate these findings and further dissect the molecular mechanisms by which ANXA1 influences SCLC’s biology.

## 5. Conclusions

Altogether, we conclude that ANXA1 is expressed in SCLC and its presence correlates with stromality and immune infiltrates, especially concerning the numbers of TAMs, where the protein expression of the molecule has been verified in situ apart from tumor cells in the TME. ANXA1-high tumors show the conspicuous enrichment of immunological pathways, mostly of an immunosuppressive nature. When studying the influence of ANXA1 silencing on the SCLC tumor cell biology, we found that, despite discrete biological changes in particular cell lines, we could not identify any unequivocal and universal effects that would warrant the development of targeted therapies against this specific molecule. While ANXA1 is an important player in SCLC’s biology, due to tumor cell heterogeneity and the adaptability of this particularly malignant cancer, its intracellular signaling is probably highly redundant and sprawling, which makes its targeting in future drug trials challenging. Despite the lack of an unambiguous downstream effect of ANXA1 silencing on SCLC cells, it might still play an important role in the TME in the establishment of an immunosuppressive microenvironment and in the recruitment of TAMs.

## Figures and Tables

**Table 1:** Multivariate Cox regression for ANXA1 expression. b = coefficient for the constant; SE = standard error around the coefficient; Wald = Wald criterion chi-squared; P = *p*-value; HR= hazard rate.

Covariate	b	SE	Wald	P	HR	95% CI of HR
ANXA1_group = 1	0.7161	0.2968	5.8199	0.0158	2.0463	1.1471 to 3.6504
Sex = 1	−1.0178	0.3953	6.6306	0.0100	0.3614	0.1672 to 0.7811
Stage_group = 1	0.5630	0.2991	3.5443	0.0598	1.7560	0.9801 to 3.1462

## Data Availability

The data that support the findings of this study are available from the corresponding author [gorbe.aniko@semmelweis.hu] upon reasonable request. Uncropped Western blot images are available in the Supplementary Materials of this article. WGS data from the UCologne2015 SCLC cohort are accessible at cBioPortal (https://www.cbioportal.org/datasets, accessed on 1 May 2024).

## Supplementary Materials

The following supporting information can be downloaded at: https://www.mdpi.com/article/10.3390/cancers17091407/s1, Figure S1. Experimental protocol and cell lines, Figure S2. Growth rate and chemosensitivity assays, Figure S3. Western blot evaluation of metabolic and mesenchymal markers, Figure S4. Western blot evaluation of dominant transcriptional drivers and immunomodulatory markers, Figure S5. Growth rate analysis in parallel with chemosensitivity assays, Supplementary Table ST1. Dilutions of antibodies in Western blot experiments, Figures S6–S14. Uncropped images of Western blots, S6. ANXA1, S7. Lactate dehydrogenase, S8. Citrate synthase, S9. ß-Catenin, S10. Vimentin, S11. CD-47, S12. PD-L1, S13. POU2F3, S14. YAP1.

## Abbreviations

The following abbreviations are used in this manuscript:

ANXA1	annexin A1
CD	cluster of differentiation
CS	citrate synthetase
LDH	lactate dehydrogenase
NSCLC	non-small cell lung cancer
OS	overall survival
PD-L1	programmed cell death ligand 1
POU2F3	POU domain, class 2, transcription factor 3
SCLC	small cell lung cancer
YAP1	yes-associated protein 1
