# Supplementary material for: Roles of Annexin A1 Expression in Small Cell Lung Cancer
Source: Cancers (Basel). 2025 Apr 23;17(9):1407. doi: 10.3390/cancers17091407 (PMC12070913; doi:10.3390/cancers17091407)
Supplement: Supplementary file 1 [file cancers-17-01407-s001.zip › cancers-3480012-supplementary.pdf]

cancers

MDPI

Supplementary Materials

Roles of Annexin A1 Expression in Small Cell Lung Cancer

Ágnes Paál, David Dora, Ákos Takács, Christopher Rivard, Shivaun Lueke Pickard, Fred R. Hirsch , Brigitta Roskó , Peter Kiraly , Péter Ferdinandy , Zoltán V. Varga , Zoltan Lohinai and Anikó Görbe

A

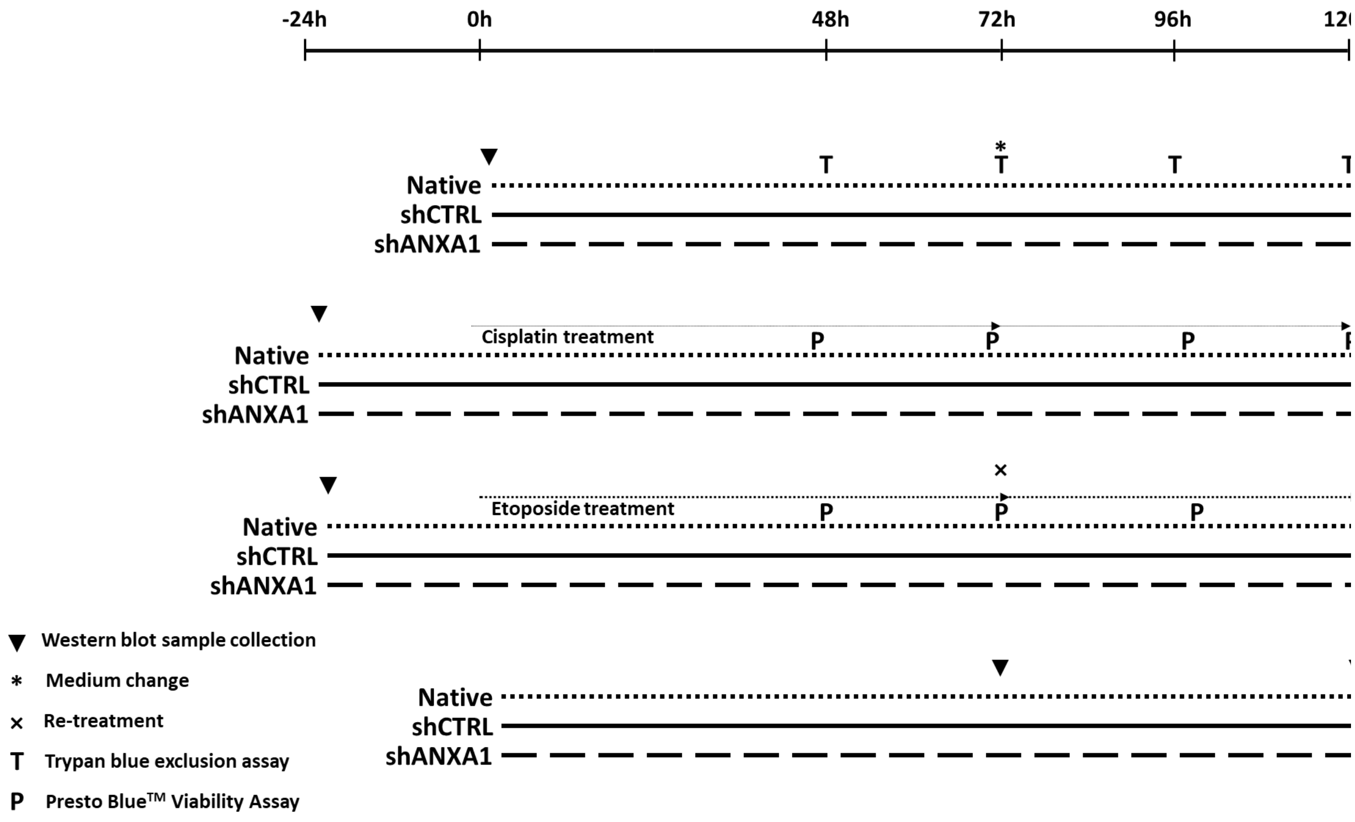

B

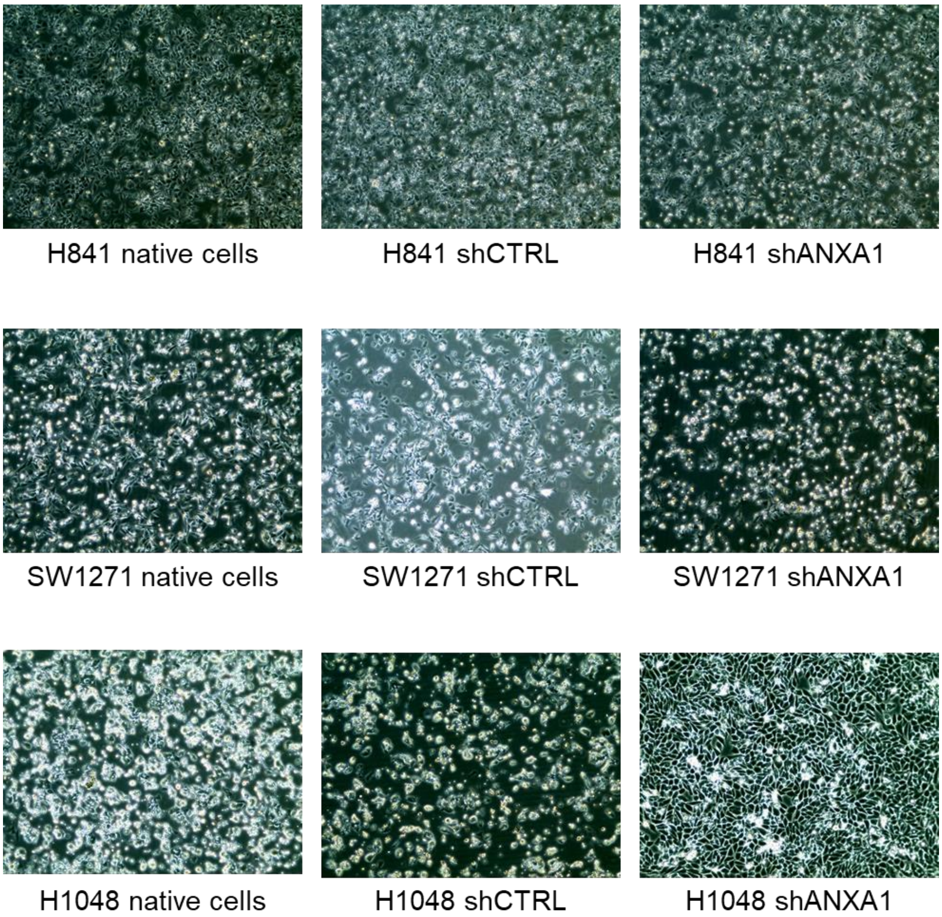

Figure S1. Experimental protocol and cell lines. A) Groups and experimental protocol. B) H841, SW1271 and H1048 cells in culture.

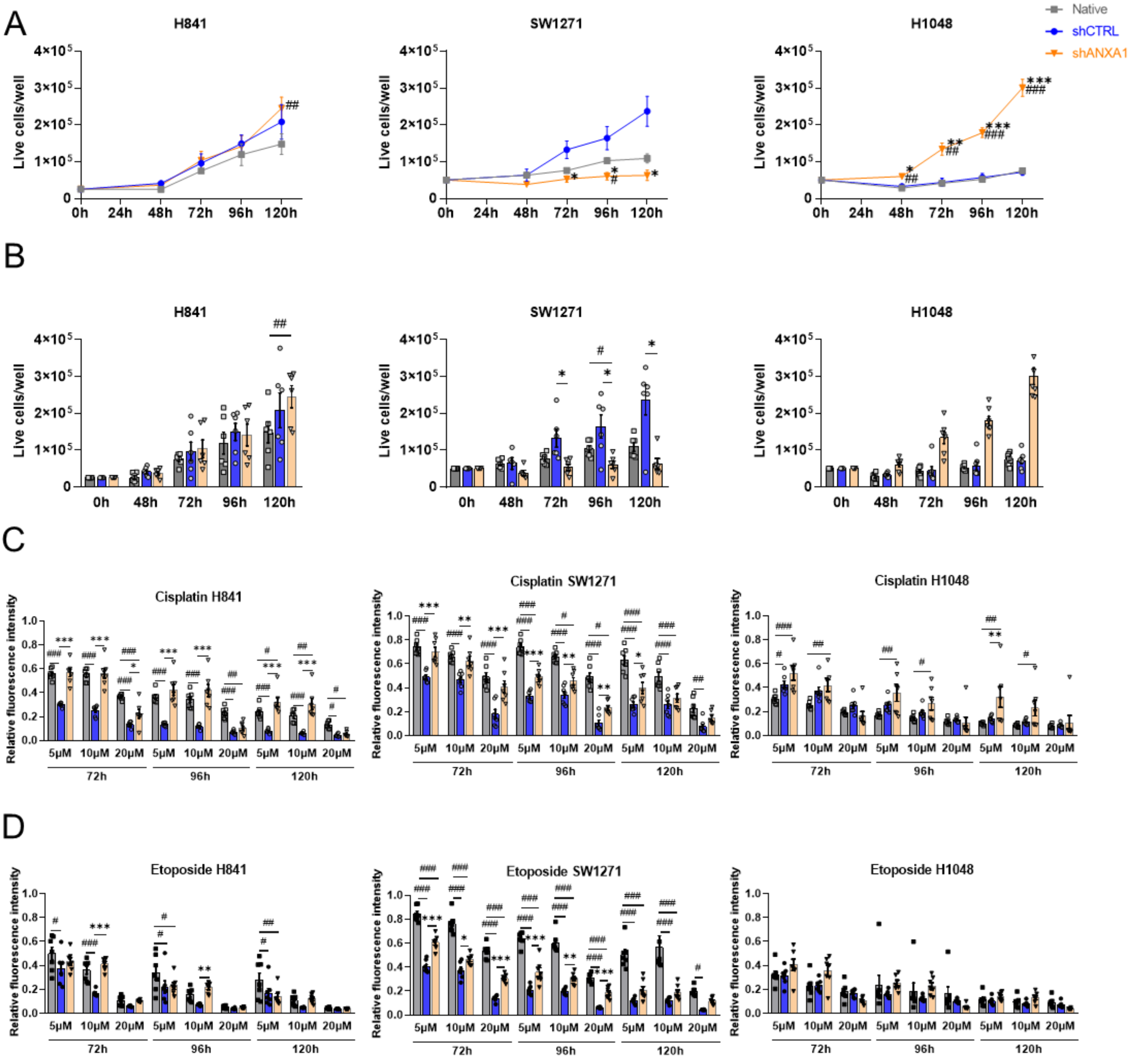

Figure S2. Growth rate and chomsensitivity assays. Growth rate assay shown as 120-hour-long growth curve **(A)** and as individual data at time points **(B).** Trypan blue exlcusion assay, n=6, one-way ANOVA, Tukey's post hoc test, $^{\#}p$<0.05, $^{\#\#}p$<0.01, $^{\#\#\#}p$<0.001: significant compared to native; **p*<0.05, ***p*<0.01, ****p*<0.001: significant compared to shCTRL. Data are displayed as mean ± SEM. Cisplatin **(C)** and etoposide **(D)** chemosensitivity assays. Presto Blue^{TM} Viability Assay, n=6-7; Two-way ANOVA, Tukey's post hoc test, $^{\#}p$<0.05, $^{\#\#}p$<0.01, $^{\#\#\#}p$<0.001: significant compared to native; **p*<0.05, ***p*<0.01, ****p*<0.001: significant compared to shCTRL. Data are displayed as mean ± SEM.

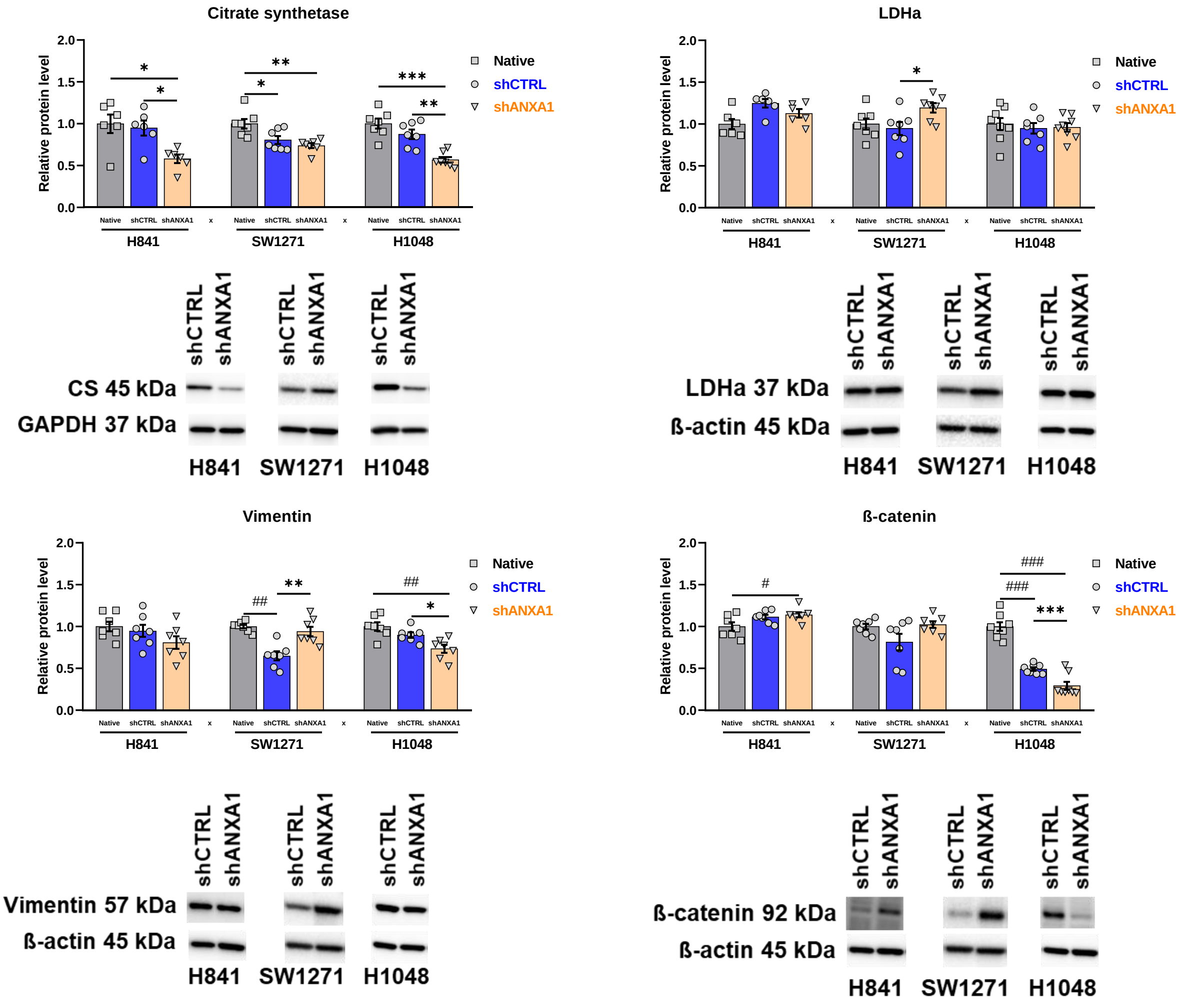

Figure S3. Western blot evaluation of metabolic and mesenchymal markers. Expression of citrate synthetase (CS) **(A)**, lactate dehydrogenase a (LDHa) **(B)**, vimentin **(C)** and ß-catenin **(D).** Western blot, n=6-8; normalized for native group of each cell line. One-way ANOVA, Tukey's post hoc test, $^{\#}p$<0.05, $^{\#\#}p$<0.01, $^{\#\#\#}p$<0.001: significant compared to native; **p*<0.05, ***p*<0.01, ****p*<0.001: significant compared to shCTRL. Data are displayed as mean ± SEM.

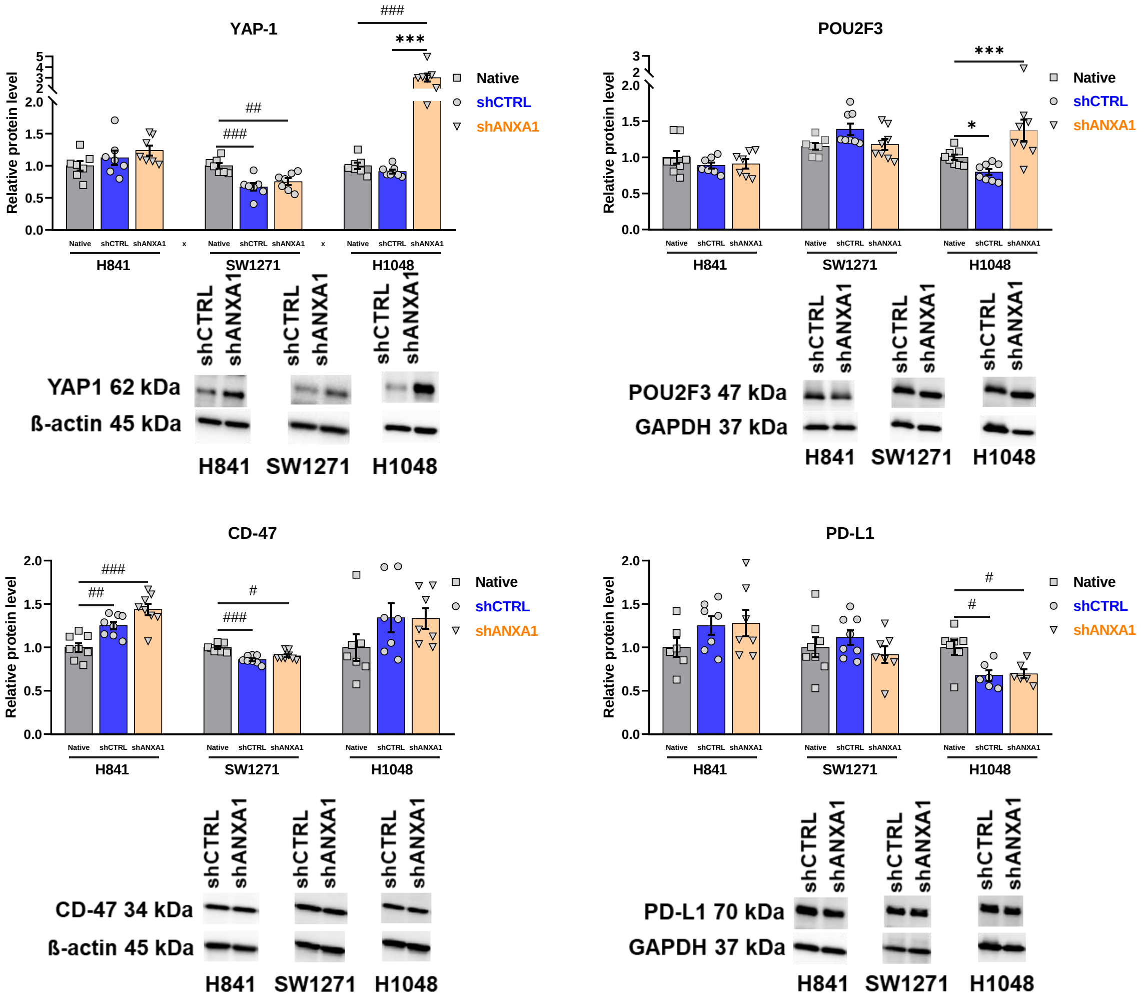

Figure S4. Western blot evaluation of dominant transcriptional drivers and immunomodulatory markers. Expression of YAP-1 **(A)**, POU2F3 **(B)**, CD-47 **(C)** and PD-L1 **(D).** Western blot, n=6-8; normalized for native group of each cell line. One-way ANOVA, Tukey's post hoc test, $^{\#}p<0.05$, $^{\#\#}p<0.01$, $^{\#\#\#}p<0.001$: significant compared to native; $^{*}p<0.05$, $^{**}p<0.01$, $^{***}p<0.001$: significant compared to shCTRL. Data are displayed as mean ± SEM.

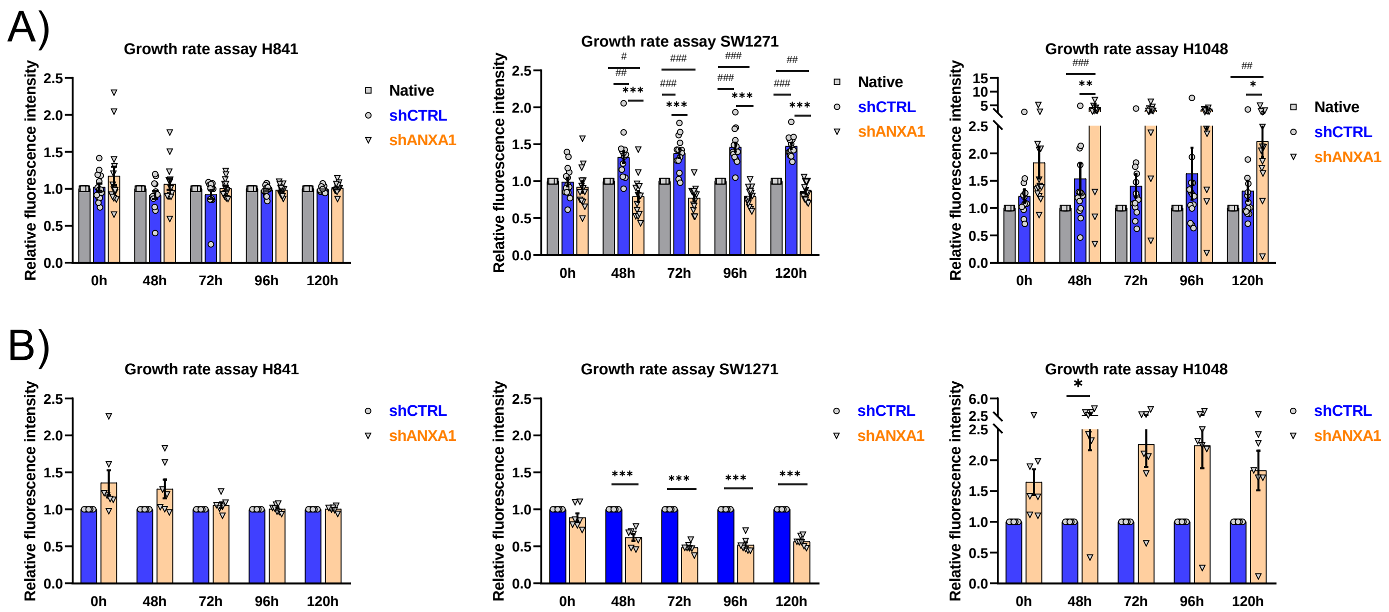

Figure S5. Growth rate analysis in parallel with chemosensitivity assays. Growth rate assay in parallel with with cisplatin and etoposide chemosensitivitiy assays, showing native group **(A).** Presto Blue™ Viability Assay, n=14, two-way ANOVA, Tukey's post hoc test, $^{\#}p<0.05$, $^{\#\#}p<0.01$, $^{\#\#\#}p<0.001$: significant compared to native; $^{*}p<0.05$, $^{**}p<0.01$, $^{***}p<0.001$: significant compared to shCTRL. Data are displayed as mean ± SEM. Growth rate assay in parallel with cisplatin and etoposide chemosensitivitiy assays showing shCTRL and shANXA1 groups **(B).** Presto Blue™ Viability Assay, n=14, one-way ANOVA, Tukey's post hoc test, $^{\#}p<0.05$, $^{\#\#}p<0.01$, $^{\#\#\#}p<0.001$: significant compared to native; $^{*}p<0.05$, $^{**}p<0.01$, $^{***}p<0.001$: significant compared to shCTRL. Data are displayed as mean ± SEM.

Table S1. Dilutions of antibodies in western blot experiments.

Primary antibodies	Dilution of primary antibodies	Dilution of secondary antibody: Anti-Rabbit IgG HRP-linked Antibody (#7074 Cell Signaling)
annexin A1 (D5V2T) XP® rabbit mAb (#32934, CellSignaling)	1:5000	1:10000
citrate synthase rabbit mAb (#ab129095, AbCam)	1:5000	1:10000
LDHA (C45B) rabbit mAb (#3582, Cell Signaling)	1:2500	1:5000
vimentin rabbit pAb (#bs-0756, Bioss)	1:2500	1:5000
ß-catenin (D10A8) XP® rabbit mAb (#8480, Cell Signaling)	1:5000	1:10000
YAP1 rabbit pAb (#PA1-46189, Invitrogen)	1:5000	1:10000
POU2F3 rabbit pAb (#av32537, Sigma Aldrich)	1:2500	1:5000
CD-47 rabbit pAb (#ab175388, Abcam)	1:2000	1:5000
PD-L1 E1L3N® XP® rabbit mAb (#13684, Cell Signaling)	1:2000	1:5000
β-actin (13E5) rabbit mAb (#4970, Cell Signaling)	1:5000	1:10000
GAPDH (D16H11) XP® rabbit mAb (#5174, Cell Signaling)	1:5000	1:10000

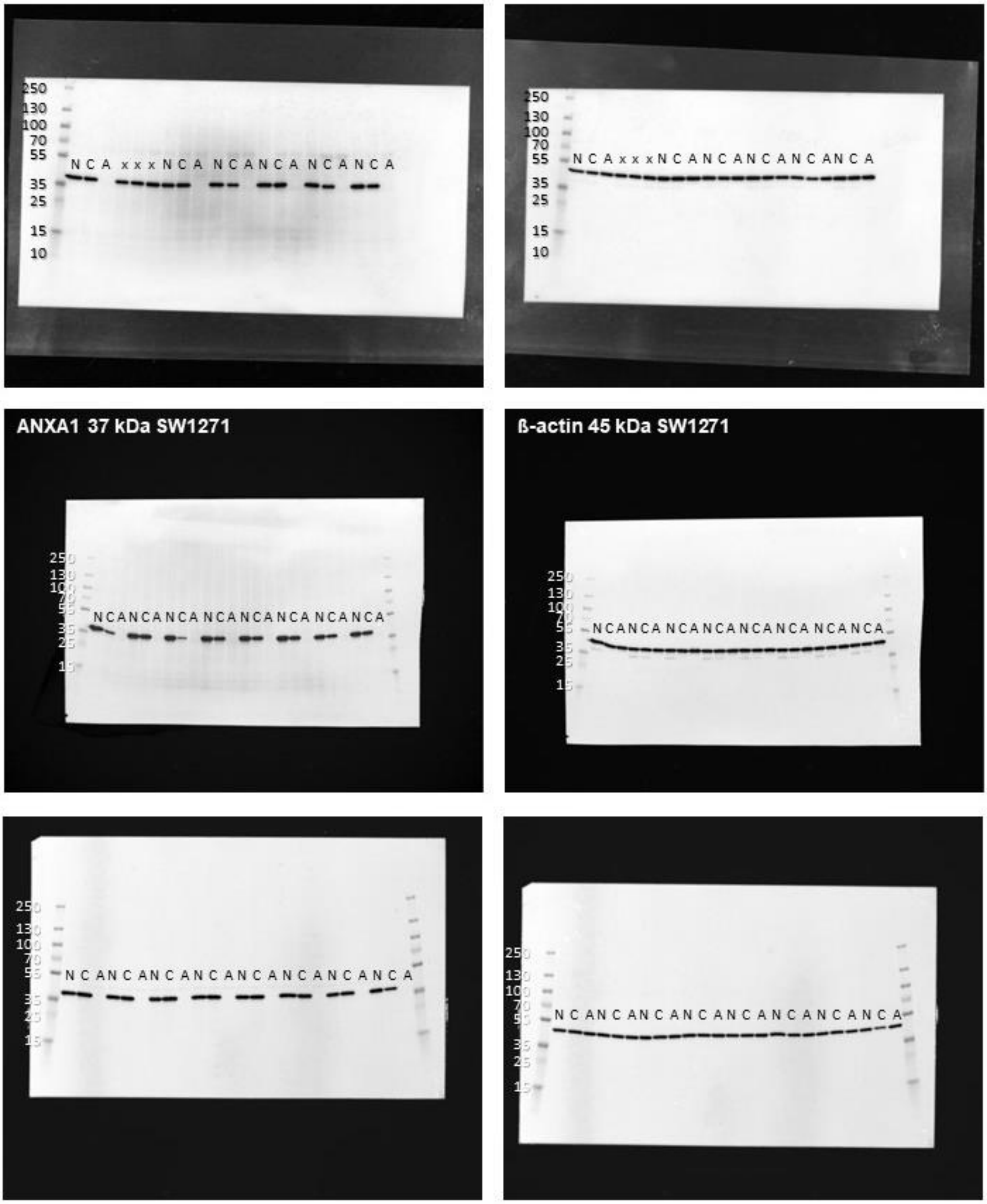

Figure S6. Uncropped images of western blots – ANXA1. N: native, C: shCTRL, A: shANXA1, x: excluded.

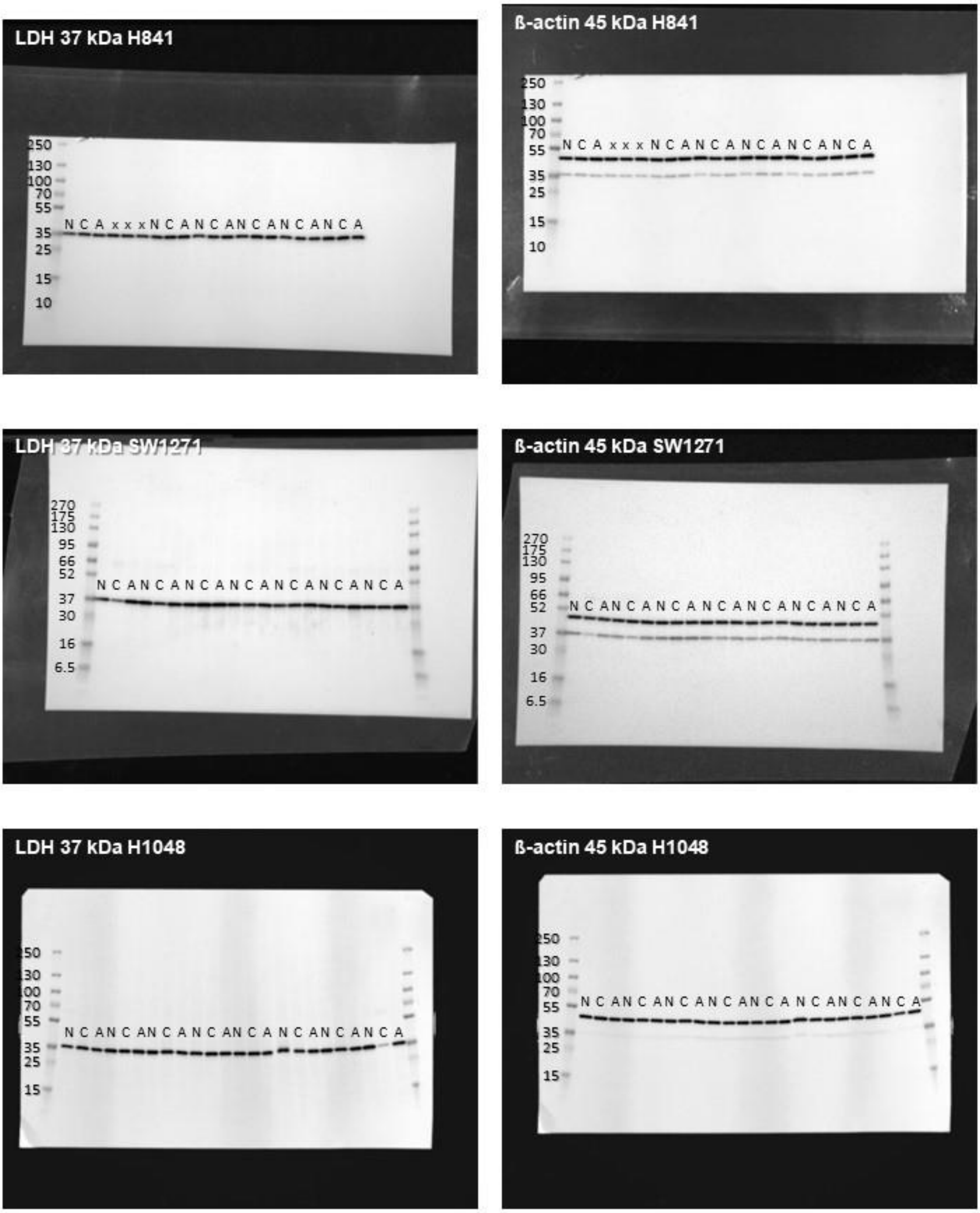

Figure S7. Uncropped images of western blots –.lactate dehydrogenase (LDH). N: native, C: shCTRL, A: shANXA1, x: excluded.

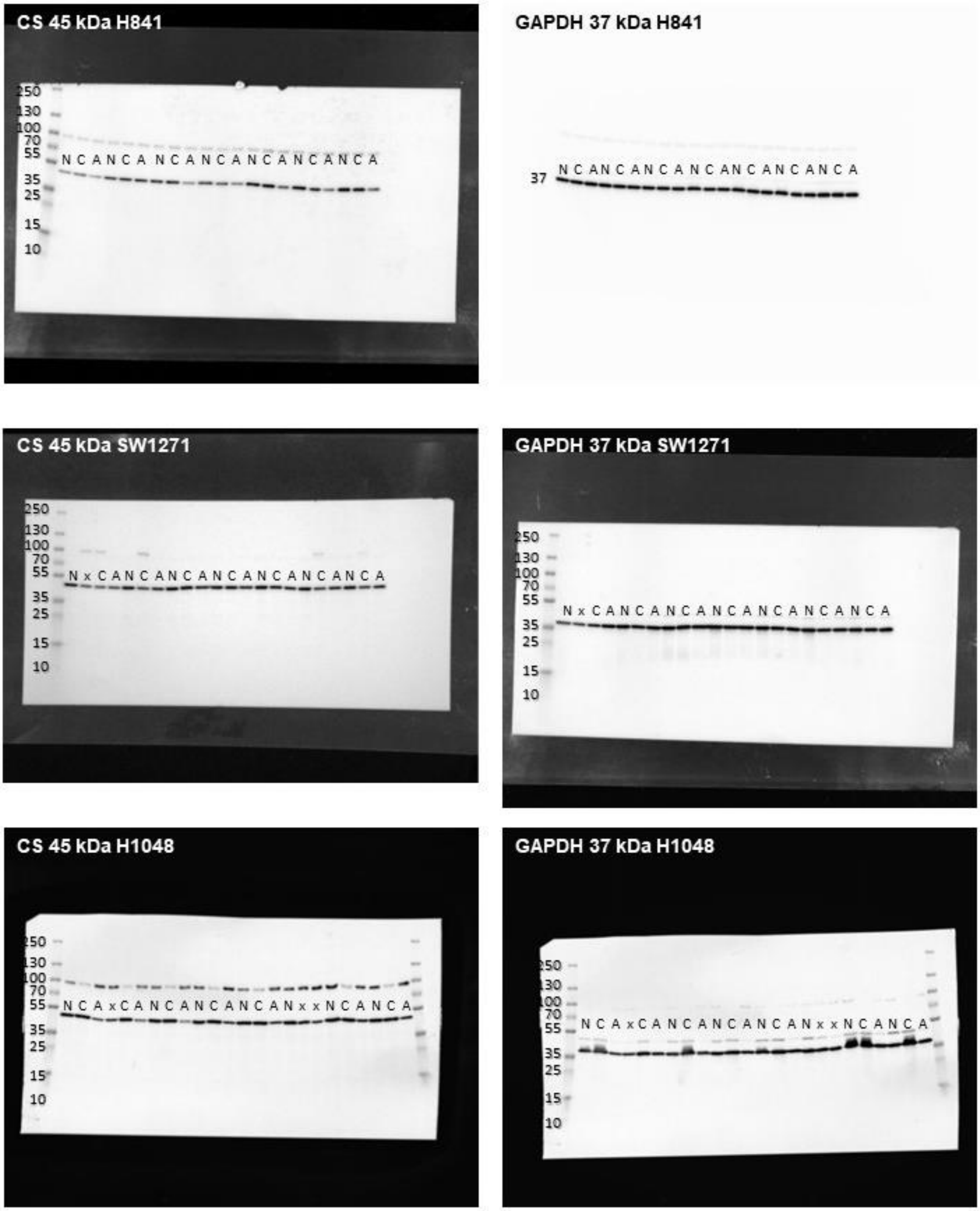

Figure S8. Uncropped images of western blots – citrate synthase (CS).

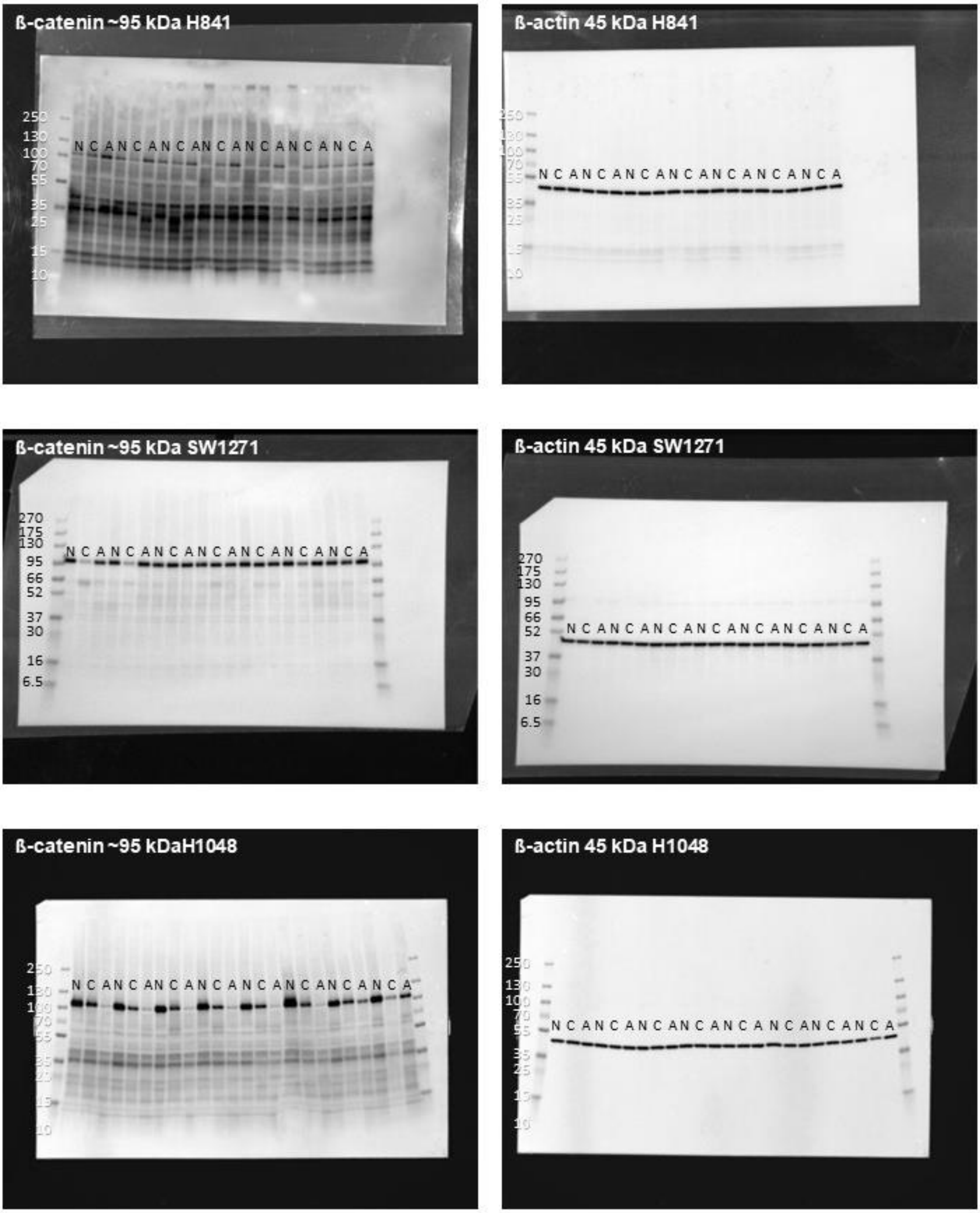

Figure S9. Uncropped images of western blots – ß-catenin. N: native, C: shCTRL, A: shANXA1.

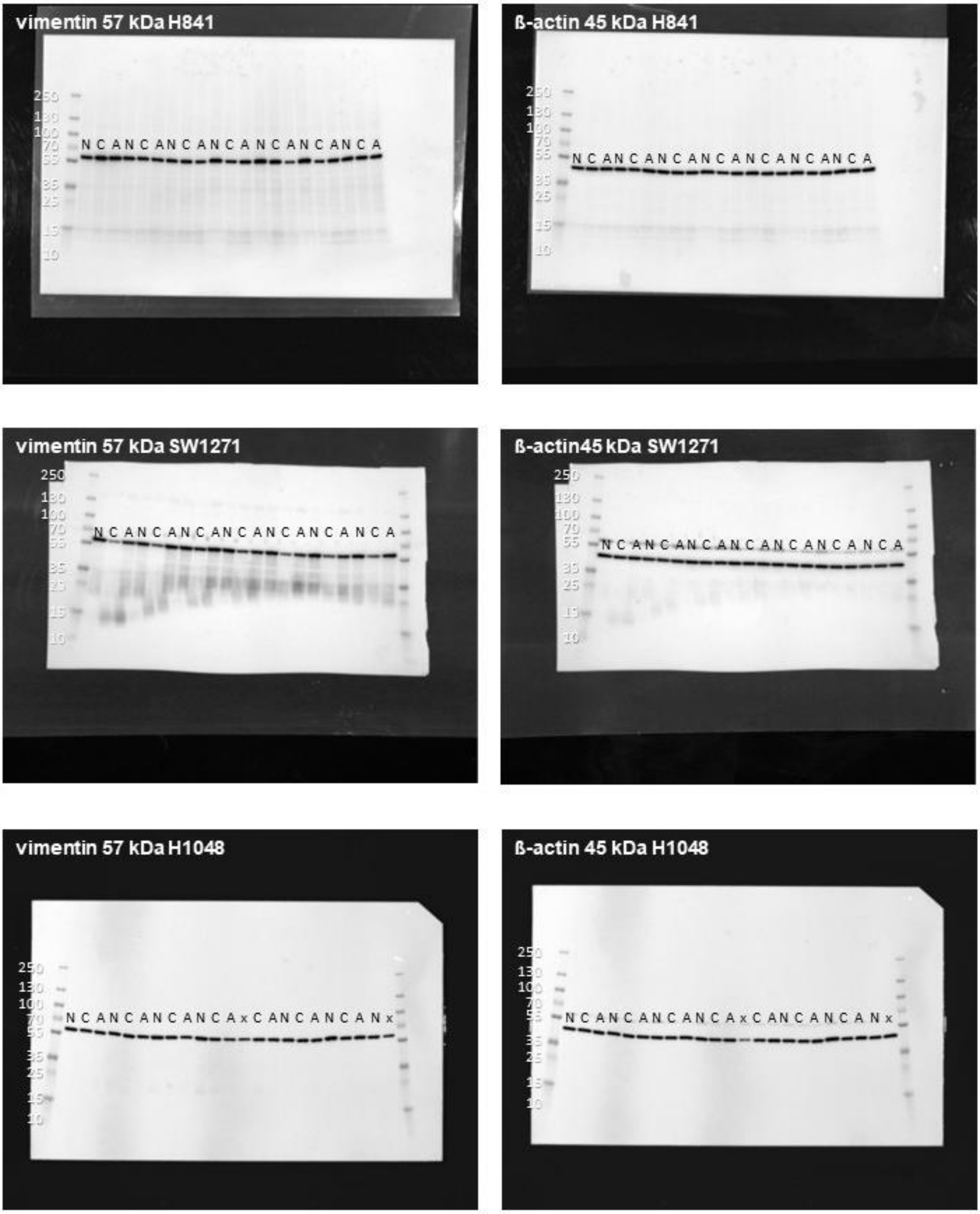

Figure S10. Uncropped images of western blots – vimentin. N: native, C: shCTRL, A: shANXA1, x: excluded.

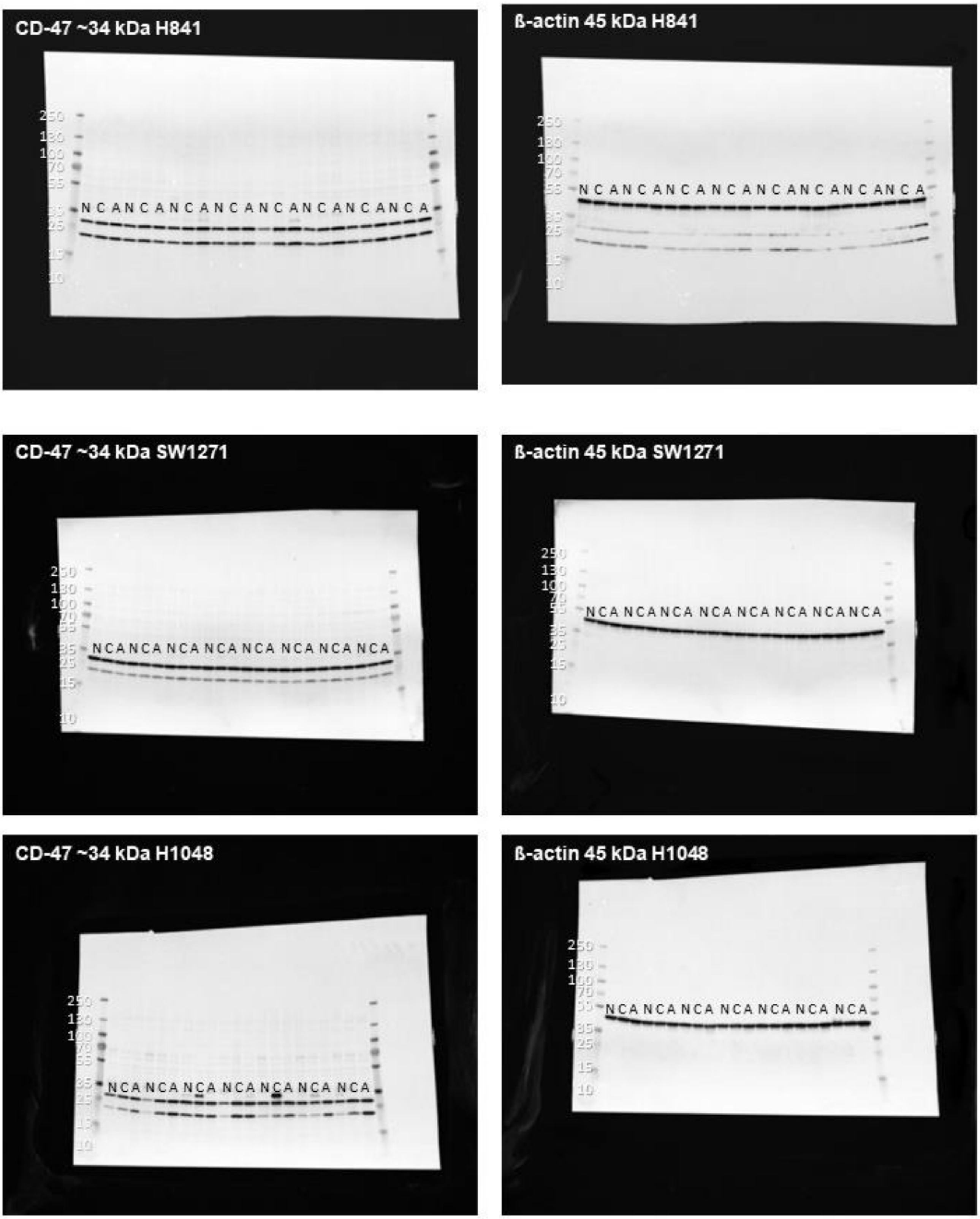

Figure S11. Uncropped images of western blots – CD-47. N: native, C: shCTRL, A: shANXA1.

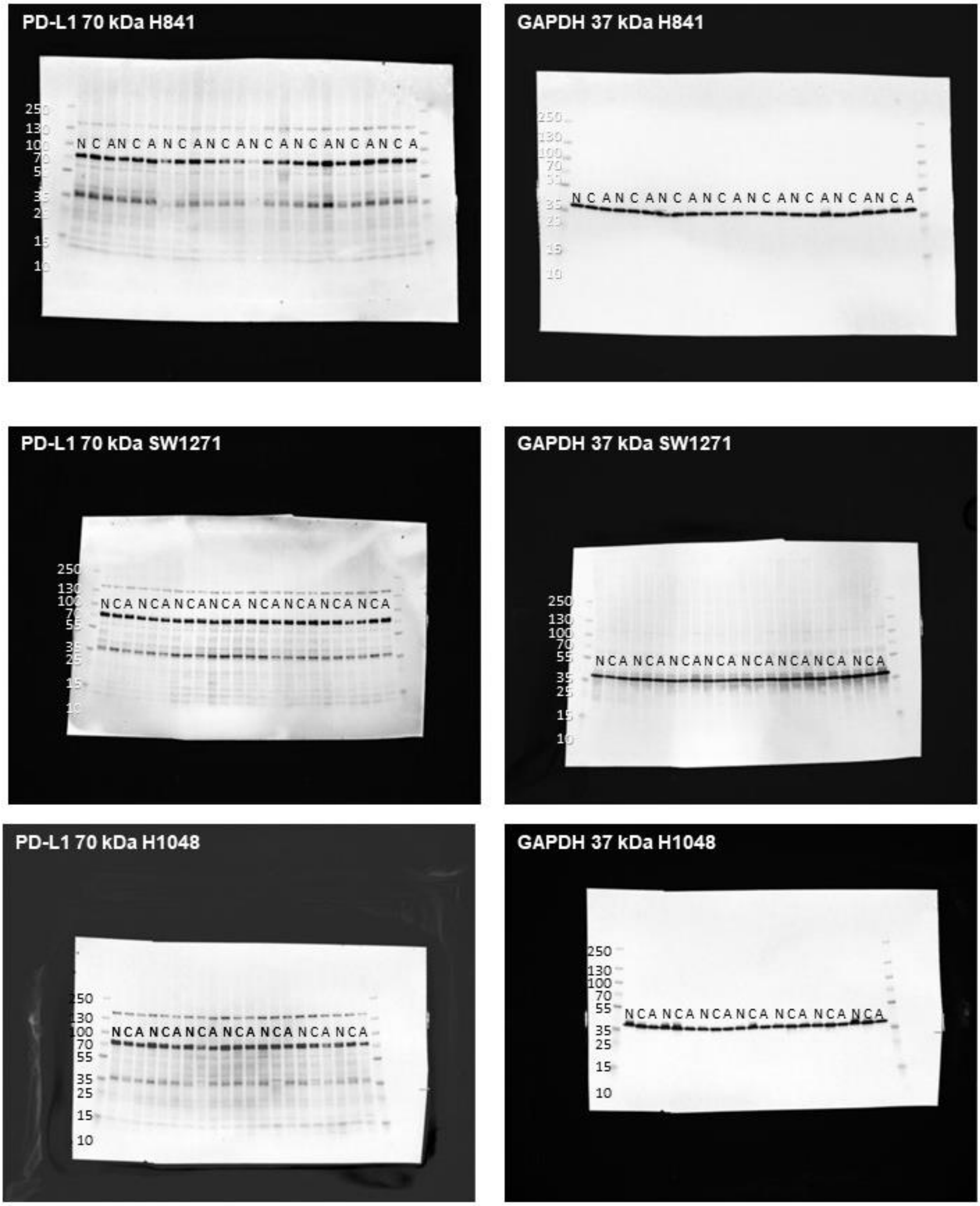

Figure S12. Uncropped images of western blots – PD-L1. N: native, C: shCTRL, A: shANXA1.

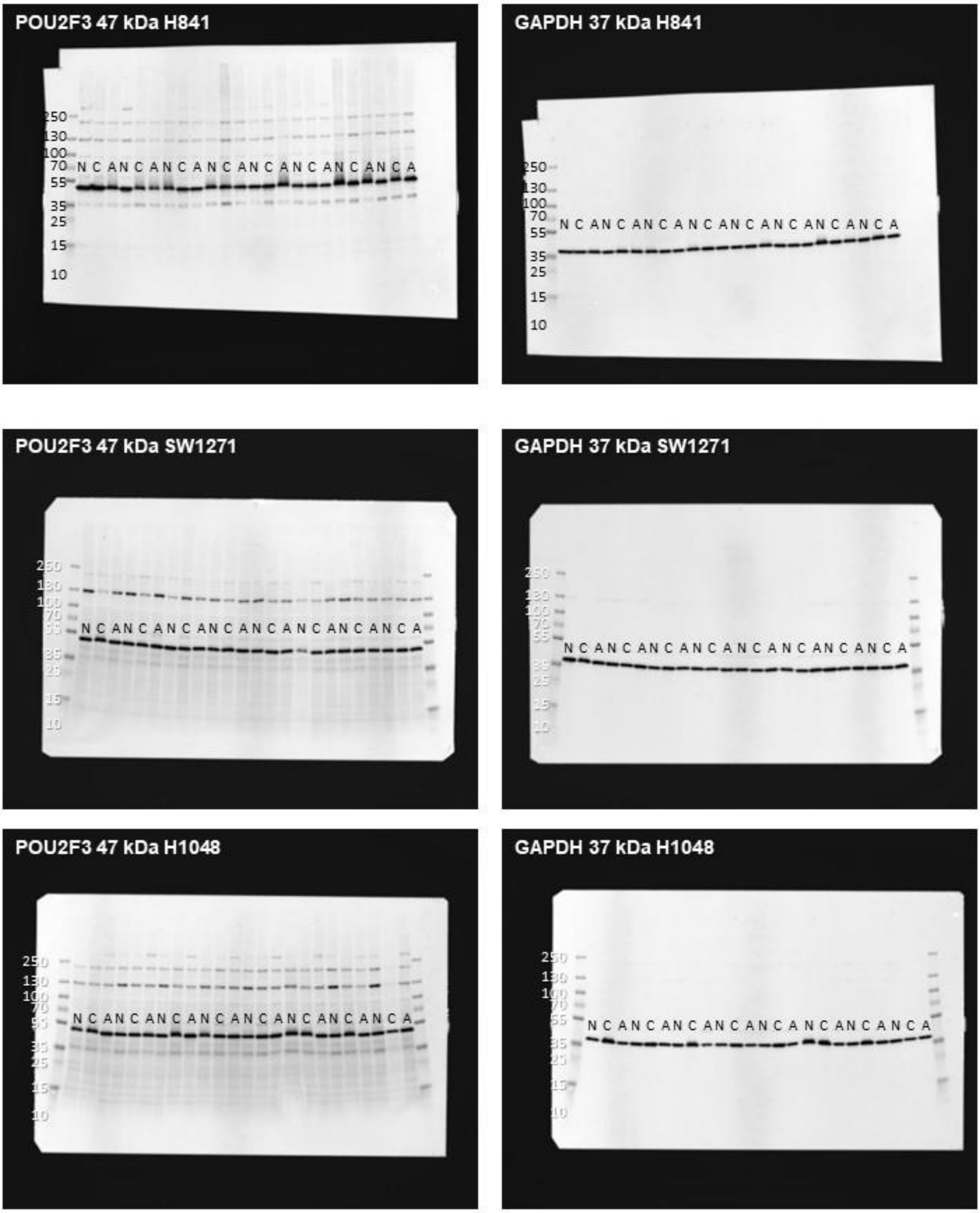

Figure S13. Uncropped images of western blots – POU2F3. N: native, C: shCTRL, A: shANXA1.

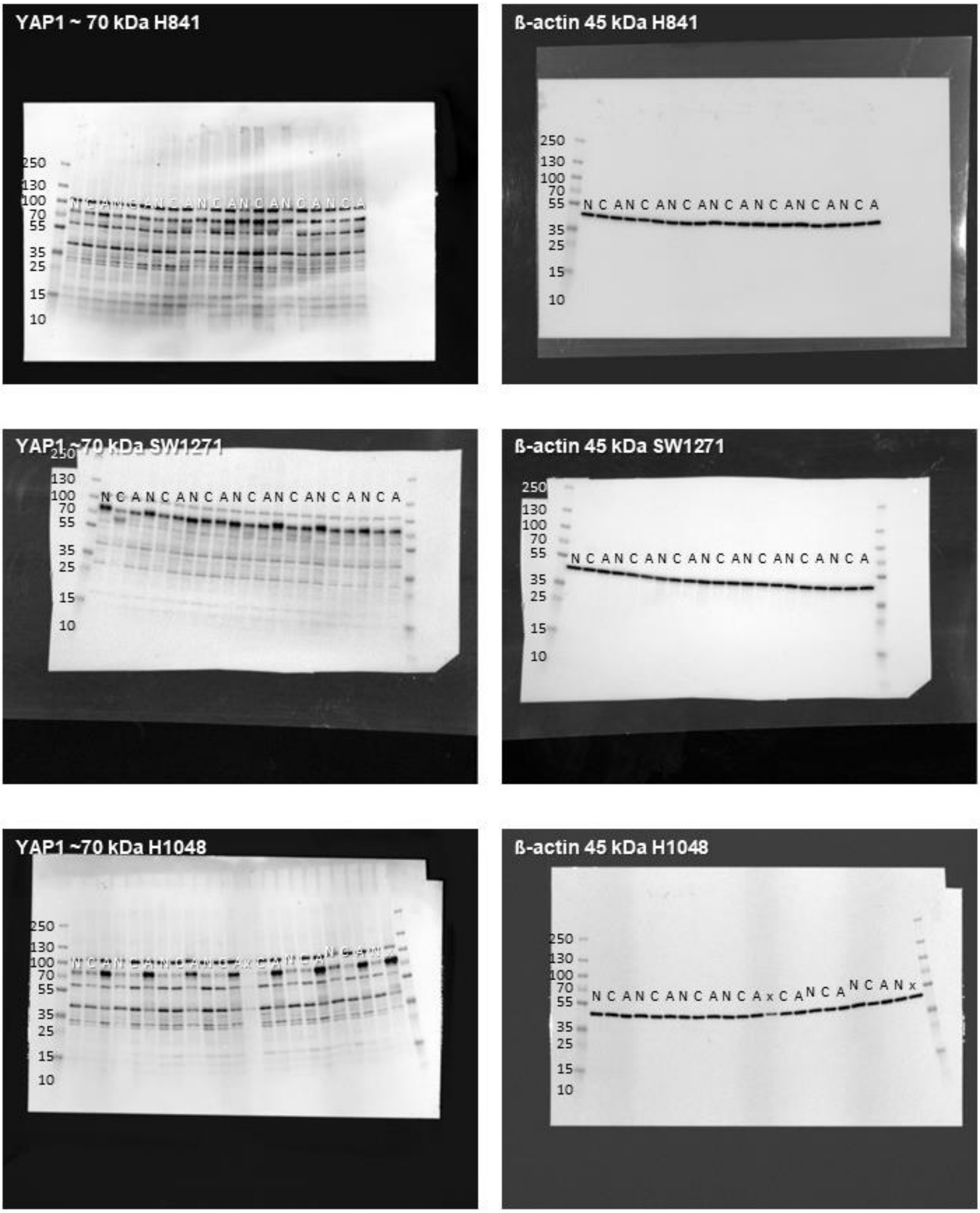

Figure S14. Uncropped images of western blots – YAP1. N: native, C: shCTRL, A: shANXA1, x: excluded.
